# Three New Cytotoxic Polyhydroxysteroidal Glycosides from Starfish *Craspidaster hesperus*

**DOI:** 10.3390/md14100189

**Published:** 2016-10-19

**Authors:** Jun-Xia Kang, Yong-Feng Kang, Hua Han

**Affiliations:** 1 School of Medicine, Tongji University, 1239 Siping Road, Shanghai 200092, China; jkang@lear.com; 2 College of Food Science and Technology, Shanghai Ocean University, 999 Huchenghuan Road, Shanghai 201306, China; yfkang@shou.edu.cn; 3 Ocean College, Zhejiang University, 1 Zheda Road, Zhoushan 316021, China

**Keywords:** starfish *Craspidaster hesperus*, polyhydroxysteroidal glycoside, cytotoxicity, tumor

## Abstract

Three new polyhydroxysteroidal glycosides, hesperuside A (**1**), B (**2**), and C (**3**), as well as a known novaeguinoside A (**4**), were isolated from the ethanol extract of starfish *Craspidaster hesperus* collected from the South China Sea. Their structures were elucidated by extensive spectroscopic methods and chemical evidence. The compounds **1**–**3** present unprecedented carbohydrate chain 3-*O*-methyl-β-d-galactopyranose, which differ from each other in the side chains. These compounds exhibited cytotoxicity against human tumor cells BEL-7402, MOLT-4, and A-549 in vitro.

## 1. Introduction

Steroidal glycosides are abundant in marine echinoderms, such as sea cucumber and starfish [1]. According to chemical structure, steroidal glycosides are subdivided into three main groups: asterosaponins, cyclic glycosides, and polyhydroxysteroidal glycosides. Polyhydroxysteroidal glycosides from starfish is one of the predominant glycosides with unique structural characteristics [2,3], which consist of a polyhydroxylated steroidal aglycone linked to one or two (rarely three) sugar units and occur in both sulfated and nonsulfated form [4,5,6]. Steroidal metabolites from starfish, especially steroidal oligoglycosides, were reported to show a broad spectrum of biological activities, including cytotoxic, hemolytic, antiviral, antibacterial, antibiofouling, neuritogenic, and antifungal effects [1,7,8,9,10]. As a continuation of our previous studies on biologically active compounds from echinoderms [11,12,13,14], we collected starfish *Craspidaster hesperus* from the South China Sea, and evaluated biological activity of the steroidal glycosides from this starfish. To our knowledge, the polyhydroxysteroidal glycosides from *C. hesperus* remains unknown, although some polyhydroxysteroidal glycosides from other starfish (e.g., *Anthenea chinensis*)*,* were reported [15]. In this study, we isolated three new polyhydroxysteroidal glycosides named hesperuside A (**1**), B (**2**), and C (**3**) from the *n*-BuOH extract of *C. hesperus*, identified the structure of these compounds, and examined their cytotoxic properties against human cancer cells.

## 2. Results and Discussion

### 2.1. Characterization of the Compounds

**Figure 1:**
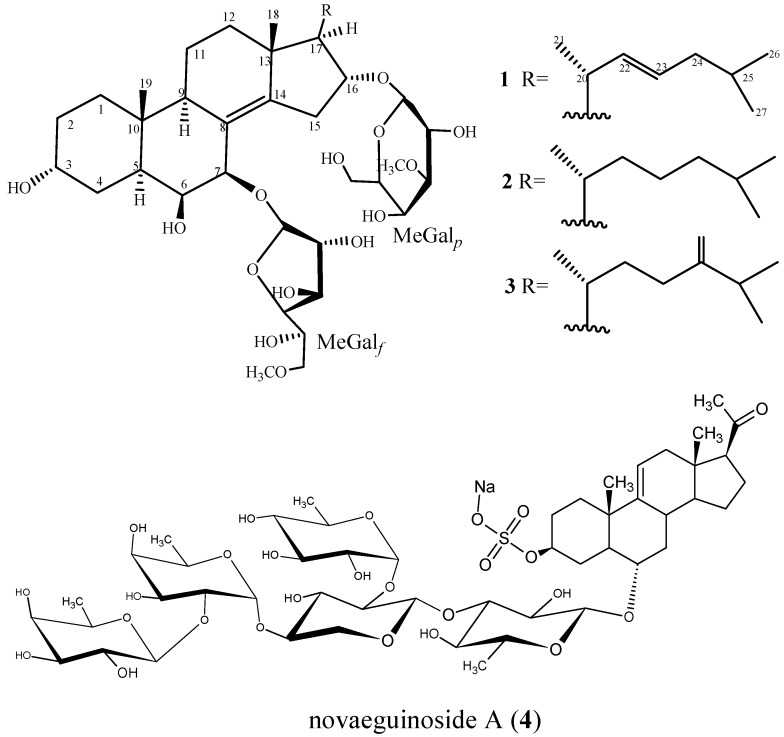
The structures of compounds **1**–**4** isolated from starfish *Craspidaster hesperus*.

The ethanolic extract from starfish *C. hesperus* was concentrated, suspended in H_2_O, and partitioned successively with petroleum ether and *n*-BuOH. The *n*-BuOH extract was subjected to several chromatographic purifications to yield three new glycosides, named as hesperuside A–C (**1**–**3**), and one known novaeguinoside A (**4**). Structures of these glycosides (Figure 1) were elucidated by extensive analysis (NMR and ESIMS) and chemical methods. 

Hesperuside A (**1**), a colorless crystalline powder, was elucidated as C_41_H_68_O_14_Na from the [M + Na]^+^ pseudomolecular ion peak at *m/z* 807.4534 (calcd. 807.4501) by the positive-ion mode HRESIMS and the [M + Na]^+^ ion peak at *m/z* 807.3 in the positive mode ESIMS. The positive results of the Liebermann–Burchard and Molisch tests suggested this compound might be a glycoside. A strong broad absorption at 3386 cm^−1^ in the IR spectrum suggested the presence of hydroxyl groups.

**Figure 2:**
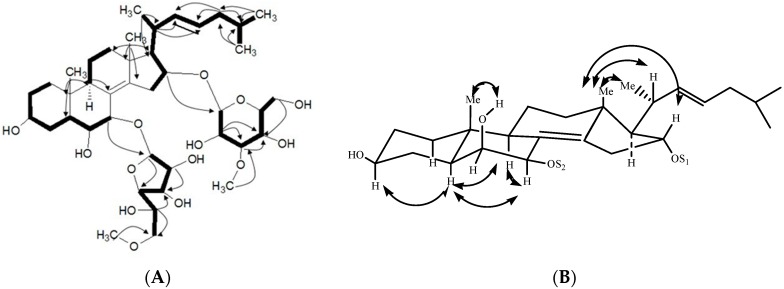
(**A**) Key correlations in the ^1^H, ^1^H-COSY (bold lines), HMBC (arrows point from protons to carbons); and (**B**) NOESY spectra of Hesperuside A (**1**).

The ^1^H- and ^13^C-NMR (DEPT) spectra data of **1** revealed the presence of a sterol aglycone that had five methyl groups (Table 1 and Table 2), including two singlets (δ_C_ 20.7, C-18 and 16.2, C-19) at δ_H_ 1.06 (s, CH_3_-18) and 1.32 (s, CH_3_-19), a doublet at δ_H_ 1.24 (d, *J* = 7.9 Hz, CH_3_-21) and the other two doublets at δ_H_ 0.96 (d, *J* = 5.1 Hz, CH_3_-26), 0.98 (d, *J* = 0.51 Hz, CH_3_-27). These data also suggested the presence of two olefinic bonds, two quaternary sp^3^ carbons at δ_C_ 39.2 (C-10), 45.0 (C-13), and two acetal methines (δ_C_ 108.5, δ_H_ 5.80 and δ_C_ 103.4, δ_H_ 4.80). These data revealed the aglycone of **1** was similar to that of anthenoside E [15], except an olefinic bond at 22(23). Resonances for one tetrasubstituted double bond at δ_C_ 127.4 (C-8), δ_C_ 146.0 (C-14), as well as one 22(23)–double bond at δ_C_ 138.0 (C-22), δ_C_ 128.6 (C-23); δ_H_ 6.15 (H-22), δ_H_ 5.45(H-23) were observed. The position of two C=C bonds in 8,14 and 22,23 was elucidated by the HMBC correlations H-6/C-8, H-7/C-8, H_3_-18/C-14, H-7/C-14, and H_3_-21/C-22, H-20/C-22, H-24/C-23, H-25/C-23, respectively. ^1^H–^1^H COSY, TOCSY, HMQC, and HMBC experiments (Figure 2) derived the assignments of the NMR signals associated with the aglycone moiety (Table 1 and Table 2). The ^13^C NMR chemical shift inventory of **1** was close to those of anthenosides J and K [15], except the signals resulted from the side chain. This deduction was supported by HMQC, HMBC, and NOESY spectra. The relative configuration of the aglycone was elucidated by analysis of NOESY data and coupling constants in pyridine-*d_5_* (Figure 2). In the NOESY spectrum, the correlation between H-5 and H-9 indicated the A/B-*trans* ring fusion. The α configuration for H-7 was deduced from the cross-peaks H-7/H-9 and H-5/H-7. The α configuration for H-6 was deduced by NOESY data and coupling constant between H-6 and H-7 (*J*_H-6/H-7_ = 2.2 Hz) [15]. The β configuration of 6-OH was deduced from the NOE cross-peaks H-5/H-6 and 6-OH/CH_3_-19. The NOE correlation between H-16 and CH_3_-18 indicated the axial orientation of H-16, so 16-OH was confirmed with the α configuration. The α configuration for H-17 and 3-OH was deduced by lack of NOE correlation between H-17 and CH_3_-18, H-3, and H-5, respectively. The NOE cross-peak CH_3_-18/H-20 and the large coupling constant *J*_H-17/H-20_ (9.9Hz), which suggested the *anti* relationship between H-17 and H-20, demonstrated the 20*R* configuration. The NOE cross-peaks H-5/H-6 and 6-OH/CH_3_-19 revealed the β configuration of 6-OH. This structure for the aglycone of **1** was confirmed by the NMR spectra and ^1^H–^1^H COSY, HMBC, HMQC, TOCSY and NOESY. Therefore, the structure of the aglycone of **1** was established as (20*R*)-5α-cholest-8(14),22(23)-diene-3α,6β,7β,16α-tetrol.

The presence of two monosaccharide units in the sugar chain of **1** was deduced from ^13^C NMR and DEPT spectra, which revealed two anomeric carbons at 108.5 and 103.4 ppm correlated by HMQC to their corresponding anomeric protons at δH 5.80 (d, *J* = 3.2 Hz) and 4.80 (d, *J* = 7.7 Hz), respectively (Table 1 and Table 2). The coupling constants of the anomeric protons were indicative in all cases of a β-configuration for the glycosidic bonds [16]. The two β-monosaccharide units were identified as galactose or its derivative by methanolysis and GC–MS analysis on the corresponding methylated hydrolysate, using the authentic samples prepared in the same manner as comparison [15,17]. The two carbohydrate units were elucidated as d-galactose by demethylation and hydrolysis with 1 N HCl. The hydrolysate was trimethylsilated, and GC retention times of the sugar were compared with authentic samples prepared in the same manner [18,19]. Comparing the NMR data with those of anthenoside J and K [15], the chemical shift at C-7 was shifted to δ_C_ 78.5 (δ_C_ 78.4 in anthenoside J and K) and the signals at C-6 and C-8 were shifted to δ_C_ 74.5 and 127.4 (δ_C-6_ 75.2 and δ_C-8_ 127.0 in anthenoside J, K), respectively, which was consistent with the linkage of sugar unit at C-7 of the aglycone. The presence of the methoxy group at C-6′ and C-3″ was revealed by the HMBC correlations from the methyl protons at δ_H_ 3.45 and 3.91 to C-6′ and C-3″ at δ_C_ 76.1 and 88.7, respectively. The ^1^H–^1^H COSY experiment elucidated the assignment of most of the resonances of each sugar ring, starting from the easily distinguished signals due to anomeric protons. Complete assignment was achieved by combining ^1^H–^1^H COSY and TOCSY results. The HMQC experiment correlated all proton resonances with those of their corresponding carbons. These data (Table 1 and Table 2) revealed that the two sugar residues were in the furanose and pyranose form (all carbon chemical shifts were downfield relative to the shifts in galactopyranose, such as δ_C-1_ < 105 and δ_C-2 and 4_ < 80 in galactopyranose [15]), respectively. The results of the GC–MS analysis, the NMR signals and comparison of the chemical shifts with those for anthenoside J and K [15] revealed that **1** had one sugar residue (6-*O*-methyl-β-d-galactofuranose, MeGal*_f_*) and the other sugar residue (3-*O*-methyl-β-d-galactopyranose, MeGal*_p_*). An HMBC experiment established the connection between the steroidal aglycone and saccharide residues at δc 78.5 (C-7) and 78.9 (C-16). The linkage of MeGal*_f_* at C-7 and MeGal*_p_* at C-16 of the aglycone was indicated by the cross-peak MeGal*_f_* H-1/aglycone C-7 and MeGal*_p_* H-1/aglycone C-16 in the HMBC spectrum, respectively. The linkage of the carbohydrate chain connected to C-7 and C-16 was confirmed by ^1^H–^1^H COSY, TOCSY, HMQC, and HMBC. Moreover, the sugar was determined as 3-*O*-methyl-d-galactopyranose, which was confirmed by the NOESY correlations of H-1′′/H-3′′, H-1′′/H-4′′ and H-1′′/H-5′′. The attachment of 3-*O*-methyl-d-Gal*_p_* moiety to C-16 of aglycone was confirmed by the correlation between H-16 and the anomeric H-1′′ in the NOESY spectrum. The ^1^H–^1^H COSY, TOCSY and HMQC experiments allowed the assignment of the proton and carbon resonances for the sugar ring. All the data indicated that hesperuside A (**1**) was (20*R*)-7-*O*-(6-*O*-methyl-β-d-galactofuranosyl)-16-*O*-(3-*O*-methyl-β-d-galactopyranosyl)-5α-cholest-8(14),22(23)-diene-3α,6β,7β,16α-tetrol. To our knowledge, few polyhydroxysteroidal glycosides with a 3α,6β,7β,16α-tetrol have been reported in starfish, and the separated MeGal*_f_* and MeGal*_p_* residues which linked at C-7 and C-16 of the aglycone have not been observed.

Hesperuside B (**2**), a colorless crystalline powder, showed positive results in the Liebermann–Burchard and Molisch tests. The molecular formula of **2** was elucidated as C_41_H_70_O_14_Na by the [M + Na]^+^ pseudomolecular ion peaks at *m/z* 809.4638 (calcd. 809.4658) in positive ion mode HRESIMS and at *m/z* 809.5 in the positive ion mode ESIMS. The IR spectrum revealed the presence of hydroxyl (3421 cm^−1^) group. The ^1^H, ^13^C-NMR, and DEPT spectroscopic data as well as the HMQC experiment (Table 1 and Table 2) revealed that the structure of **2** is similar to that of **1**, except the signals of the side chain. The NMR spectroscopic data showed that the steroidal aglycone of **2** is same to that of anthenoside E [15]. The ^1^H NMR data showed that **2** contains two methyl singlets at δ_H_ 1.06 (s, CH_3_-18) and 1.32 (s, CH_3_-19), one methyl doublets at δ_H_ 1.09 (d, *J* = 7.2 Hz, CH_3_-21), two methyl groups at δ_H_ 0.92 (CH_3_-26 and CH_3_-27) with same coupling constant *J* = 7.6, three methylene, and one methylidyne. The cross-peaks H-20/H-21, H-20/H-22, H-22/H-23, H-23/H-24, H-25/H_3_-26, H-25/H_3_-27 and H-25/H-24 in ^1^H–^1^H COSY spectra revealed the difference at C-22, C-23 between **2** and **1**. This result was confirmed by TOCSY, HMQC, HMBC and NOESY. A comparison on the NMR data of the carbohydrate chain of **1** and **2** showed that these compounds had the same saccharide chain. Thus, the structure of **2** was deduced as (20*R*)-7-*O*-(6-*O*-methyl-β-d-galactofuranosyl)-16-*O*-(3-*O*-methyl-β-d-galactopyranosyl)-5α-cholest-8(14)-en-3α,6β,7β,16α-tetrol.

**Figure 3:**
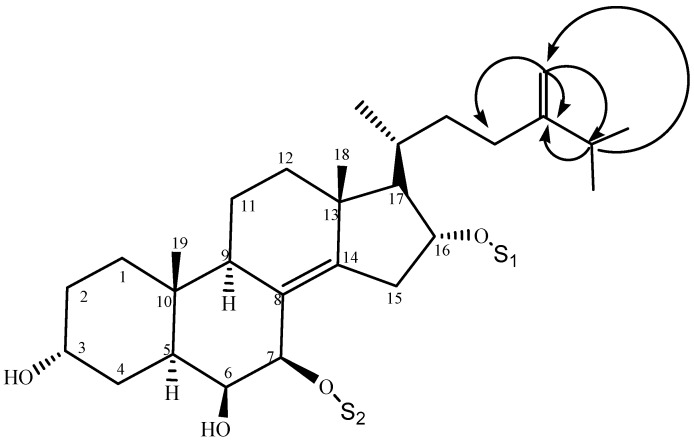
Key HMBC correlations of the side chain of hesperuside C (**3**).

Hesperuside C (**3**), a white crystalline powder, showed [M + Na]^+^ pseudomolecular ion peak at *m/z* 821.4654 in the positive-ion mode HRESIMS, which was consistent with the molecular formula C_42_H_70_O_14_Na. The results of Liebermann–Burchard and Molisch tests were positive. The NMR data (Table 1 and Table 2) revealed that the aglycone of **3** was same to that of anthenoside G [15]. The ^1^H, ^13^C NMR and DEPT spectra data of **3** as well as HMQC experiment revealed the presence of one tetrasubstituted double bond (δ_C_ 126.3, C-8 and 146.5, C-14), two quaternary sp^3^ carbons (δ_C_ 38.6, C-10 and 44.5, C-13), one terminal double bond (δ_C_ 157.1, C-24; 107.5, C-28, and δ_H-28_ 4.75, 4.77), two acetal methines (δ_C_ 108.3, δ_H_ 5.61 and δ_C_ 102.9, δ_H_ 4.70), and five methyl groups (Table 1 and Table 2). Comparison of the NMR and DEPT spectroscopic data revealed that **2** and **3** have the same steroidal skeleton, except the side chain. The chemical shift of C-24 in **3** was shifted downfield to δ_C_ 157.1, indicating the terminal double bond at C-24. This deduction was confirmed by ^1^H–^1^H COSY, HMQC and HMBC spectra. HMBC correlations from H_2_-28 to C-23, C-24, and C-25 and from H-25 to C-24, and C-28 were observed (Figure 3). Thus, the structure of **3** was deduced as (20*R*)-7-*O*-(6-*O*-methyl-β-d-galactofuranosyl)-16-*O*-(3-*O*-methyl-β-d-galactopyranosyl)-5α-cholest-8(14),24(28)-diene-3α,6β,7β,16α-tetrol.

### 2.2. Cytotoxic Activities

The cytotoxic activities of glycosides **1**, **2**, and **3** were evaluated against human leukemia MOLT-4, human hepatoma BEL-7402, and human lung cancer A-549 cell lines. HCP (10-hydroxycamptothecin) served as the positive control. The compound **2** exhibited cytotoxicity against all the tested human tumor cells, while **1** and **3** were partially active against A-549 and BEL-7402 cells (Table 3).

Previous studies reported that cytotoxic activity varied among the steroidal glycosides from starfish, and indicated that the structural feature of the steroidal glycosides is responsible for their cytotoxic activity [15,20,21]. The saccharide units and their position of attachment on the aglycone, as well as the side chains, were assumed to be important for inhibiting the proliferation of the human cancer cells. Results of our study are consistent with the viewpoints. In addition, our study suggests the potential of **2** as anticancer lead compounds. The structure–function relationships for these steroidal glycosides are the subject of ongoing studies.

## 3. Experimental Section

### 3.1. General Experimental Procedures

Optical rotations were measured with Perkin-Elmer 341 polarimeter. IR spectra were recorded on a Bruker Vector-22 infrared spectrometer in cm^−1^. NMR spectra were recorded in C_5_D_5_N on a Varian Inova-600 spectrometer at 600 MHz (^1^H) and 150 MHz (^13^C) (tetramethylsilane was used as an internal standard), and 2D NMR spectra were obtained using standard pulse sequences. Coupling constants (*J*) are given in Hz. ESIMS and HRESIMS were obtained on a Micromass Quattro mass spectrometer. The methylated hydrolysates were analyzed with GC–MS using an Agilent 6890 GC/5973 MS with an HP-1 column (30 m × 0.32 mm i.d.). The trimethylsilated hydrolysates were analyzed with GC using a Finnigan Voyager apparatus with a 1-Chirasil-Val column (25 m × 0.32 mm i.d., 0.25 μm). Semipreparative HPLC was conducted using an Agilent 1100 liquid chromatograph equipped with a refractive index detector using a Zorbax 300 SB-C_18_ column (5 μm, 250 × 9.4 mm i.d., Agilent, CA, USA). Column chromatographies were performed on silica gel H (200–300 mesh, 10–40 μm, Qingdao Marine Chemical Inc., Qingdao, China), reversed-phase silica gel (Lichroprep RP-18, 40–63 μm, Merck Inc., Shanghai, China) and Sephadex LH-20 (Pharmacia, Beijing, China). Fractions were monitored by TLC precoated silica (Si) gel HSGF_254_ plates (CHCl_3_–EtOAc–MeOH–H_2_O, 4:4:2.5:0.5) or RP-C18 (MeOH–H_2_O, 1:1) that were purchased from Qingdao Marine Chemical Inc. (Qingdao, China).

### 3.2. Animal Material

The starfish was collected from the South China Sea (Shantou, Guangdong Province, China) in June 2010. The organism was identified as *C. hesperus* by Prof. Y.-L. Liao (the Institute of Oceanology, Chinese Academy of Science, Qingdao, China). A voucher specimen (No. SA201006) was preserved in the Ocean College of Zhejiang University (Zhoushan, China).

### 3.3. Extraction and Isolation

The starfish (1.5 kg, dried) were chopped, and extracted with 65% EtOH (3 L) four times (1 h for each time) under reflux. The extracts were concentrated to leave a reddish residue. The residue was dissolved in H_2_O (2 L), and then partitioned successively using petroleum ether (2 L × 4) and *n*-BuOH (2 L × 5). The *n*-BuOH fraction (19 g) was chromatographed over silica gel column (500 g, 3× 100 cm) eluting with CHCl_3_/MeOH/H_2_O (stepwise gradient 10:1:0 to 6:4:0.5), and 10 major fractions (A–J) were obtained based on TLC analysis. Fraction I (1200 mg) was chromatographed on reversed phase silica MPLC (Lichroprep RP-C_18_, 40–63 μm, 2.5 cm × 50 cm) eluting with MeOH/H_2_O (stepwise gradient 30:70 to 90:10) and subjected to Sephadex LH-20 column (2 cm × 120 cm) equilibrated MeOH/H_2_O (2:1). Finally, fraction I-1 (120 mg) was purified by semipreparative HPLC (Zorbax 300 SB-C18; 85% aq. MeOH, 1.5 mL/min) to afford pure glycosides hesperuside A (**1**) (26.2 mg; *t*_R_ 18.89 min), B (**2**) (36 mg; *t*_R_ 20.01min), and C (**3**) (18.5 mg; *t*_R_ 21.06 min). Fraction D (550 mg) was subjected to MPLC on a Lobar column (Lichroprep RP-18, 40–63 μM, 2 × 50 cm) with MeOH/H_2_O (65:35) as eluent system, and then was subjected to size exclusion chromatography on a Sephadex LH-20 column (2 cm × 120 cm) eluting with MeOH/H_2_O (80:20). Finally, fraction D was purified by HPLC (Zorbax 300 SB-C18, 5 μM; 250 mm × 9.4 mm i.d.; 47% aq. MeOH, 1.5 mL/min) to afford compound **4** (36 mg, *t*_R_ 36.70 min).

Hesperuside A (**1**): C_41_H_68_O_14_Na, colorless crystalline powder; ${\left[\alpha \right]}_{D}^{20}$: −51.04 (*c* 1, MeOH); ^1^H and ^13^C NMR data are shown in Table 1 and Table 2; ESIMS (pos.) *m/z* 807.3 [M + Na]^+^; HRESIMS (pos.) *m/z* 807.4534 [M + Na]^+^ (calcd. for C_41_H_68_O_14_Na^+^, 807.4501).

Hesperuside B (**2**): C_41_H_70_O_14_Na, colorless crystalline powder; ${\left[\alpha \right]}_{D}^{20}$: −64.69 (*c* 1 MeOH); ^1^H and ^13^C NMR data are shown in Table 1 and Table 2; ESIMS (pos.) *m/z* 809.5 [M + Na]^+^, 647.6 [M + Na − 62]^+^; HRESIMS (pos.) *m/z* 809.4638 [M + Na]^+^ (calcd. for C_41_H_70_O_14_Na^+^, 809.4658).

Hesperuside C (**3**): C_42_H_70_O_14_Na, white crystalline powder; ${\left[\alpha \right]}_{D}^{20}$: −47.36 (*c* 1, MeOH); ^1^H and ^13^C NMR data are shown in Table 1 and Table 2; ESIMS *m/z* 821.5 [M + Na]^+^; HRESIMS *m/z* 821.4748 [M + Na]^+^ (calcd. for C_42_H_70_O_14_Na, 821.4768).

### 3.4. Methanolysis of ***1***–***3*** [15]

Each sample (1 mg) was resolved with 2 M HCl/MeOH (0.5 mL) and heated at 80 °C for 12 h. The reaction mixture was evaporated to dryness, and the residue was partitioned between EtOAc and H_2_O. The aqueous phase was concentrated under reduced pressure. DMSO (0.3 mL) and 50% aqueous sodium hydroxide (0.03 mL) were added to the dried residue, and the mixture was stirred vigorously. Iodomethane (0.05 mL) was immediately added dropwise, and the mixture was further stirred for 4 h. The suspension was then poured into water (0.5 mL) and extracted with CH_2_Cl_2_ (4 mL × 3). The CH_2_Cl_2_ extracts were washed with water and dried over sodium sulfate, and then concentrated under reduced pressure. The resulting products and the standard sugar derivatives prepared under the same conditions were analyzed by GC–MS (initial temperature was 100 °C for 2 min, and then the temperature was increased to 240 °C at a rate of 8 °C/min). The derivatives of d-glucose and d-galactose were detected with *t*_R_ of 11.91 and 12.85 min, respectively. All the compounds gave peaks of the derivative of d-galactose.

### 3.5. Demethylation and Acid Hydrolysis of ***1***–***3*** [15]

For demethylation of **1**, **2,** and **3**, each sample (1 mg) was mixed with 1 mL of dry dichloromethane and 0.01 mL of boron tribromide at −80 °C for 30 min, and then stood overnight at 10 °C under anhydrous condition. The solvent and reagent were evaporated to dryness in vacuo at room temperature. The demethylated derivative of each sample was heated with 1 mL of 2 M CF_3_COOH at 120 °C for 2 h. The reaction mixture was evaporated under vacuum, and the residue was partitioned between CH_2_Cl_2_ and H_2_O. The aqueous phase was concentrated and dissolved in 1-(trimethylsilyl) imidazole and anhydrous pyridine (0.1 mL). Then, the solution was stirred at 60 °C for 5 min and dried with a stream of N_2_. The residue was partitioned between CH_2_Cl_2_ and H_2_O. The CH_2_Cl_2_ layer was analyzed by GC (initial temperature was 100 °C for 1 min, and the temperature was increased to 180 °C at a rate of 5 °C/min). The peaks of the derivatives of the samples were detected at 13.81 and 14.79 min. Retention times for authentic samples after being treated simultaneously with 1-(trimethylsilyl) imidazole in pyridine were 13.80 and 14.78 min (d-galactose) and 13.62 and 14.59 min (l-galactose), respectively.

### 3.6. Cytotoxicity Assay

The cytotoxicity of glycosides **1**–**3**, against human leukemia MOLT-4, human lung cancer A-549, and human hepatoma BEL-7402 cells (Shanghai Institute of Materia Medical, Chinese Academy of Sciences) was evaluated by the sulforhodamine B (SRB) protein assay following the method described in [22]. The anticancer agent 10-hydroxycamptothecin served as a positive control. Dose–response curves were plotted for the samples, and the IC_50_ values were defined as the concentrations of the test glycosides at which 50% reduction of absorption was observed. The data represented the means of three independent experiments in which each compound concentration was tested in three replicate wells. The concentration inducing 50% inhibition of cell growth (IC_50_) was determined graphically for each experiment by curve-fitting using Prism 4.0 software (GraphPad software, Inc., Shanghai, China) and the equation derived by De Lean et al. [23]. The IC_50_ value for each treatment (**1**, **2**, **3**, and the control) was expressed as mean ± S.D. (*n* = 3), and the difference in the IC_50_ value between the tested compound (**1**, **2**, or **3**) and control was examined using Student’s-test. *P* < 0.05 was accepted as significant difference.

## 4. Conclusions

Three new polyhydroxysteroidal glycosides (**1**–**3**) along with a known one (**4**) were isolated from the alcoholic extract of starfish *C. hesperus*, and their chemical structures were elucidated by extensive NMR and ESIMS techniques. Hesperuside A–C (**1**–**3**) contained the unprecedented carbohydrate chain 3-*O*-methyl-β-d-galactopyranose and nonsulfate in the sugar chain. The linkage of two carbohydrate chains connected to C-7 and C-16, respectively. This study reveals that glycosides of starfish could be a source of anticancer natural products and warrant further biomedical investigation since hesperuside A–C (**1**–**3**) exhibited moderate cytotoxic activity against BEL-7402, MOLT-4, and A-549 cells.

## Figures and Tables

**Table 1:** ^13^C NMR spectroscopic data (150 MHz, pyridine-*d*_5_) for hesperusides A–C (**1**–**3**) ^1^ (δ in ppm).

Position	1	2	3
1	34.6, CH_2_	34.4, CH_2_	33.8, CH_2_
2	30.5, CH_2_	30.5, CH_2_	29.6, CH_2_
3	66.7, CH	66.7, CH	65.7, CH
4	34.3, CH_2_	34.3, CH_2_	33.7, CH_2_
5	38.5, CH	38.4, CH	37.5, CH
6	74.5, CH	74.6, CH	78.0, CH
7	78.5, CH	78.8, CH	76.9, CH
8	127.4, qC	127.2, qC	126.3, qC
9	46.0, CH	45.7, CH	44.9, CH
10	39.2, qC	39.2, qC	38.6, qC
11	19.5, CH_2_	19.7, CH_2_	18.6, CH_2_
12	36.7, CH_2_	37.3, CH_2_	36.7, CH_2_
13	45.0, qC	45.0, qC	44.5, qC
14	146.0, qC	147.1, qC	146.5, qC
15	34.5, CH_2_	34.7, CH_2_	34.5, CH_2_
16	78.9, CH	79.6, CH	77.8, CH
17	62.1, CH	62.2, CH	61.2, CH
18	20.7, CH_3_	20.3, CH_3_	19.3, CH_3_
19	16.2, CH_3_	16.2, CH_3_	15.4, CH_3_
20	36.2, CH	33.6, CH	33.2, CH
21	24.7, CH_3_	21.2, CH_3_	21.9, CH_3_
22	138.0, CH	35.4, CH_2_	25.0, CH_2_
23	128.6, CH	25.9, CH_2_	33.7, CH_2_
24	42.9, CH_2_	40.3, CH_2_	157.1, qC
25	29.5, CH	28.9, CH	35.8, CH
26	23.2, CH_3_	23.2, CH_3_	23.9, CH_3_
27	23.2, CH_3_	23.2, CH_3_	23.9, CH_3_
28			107.5, CH_2_
MeGal*_f_*			
1′	108.5, CH	108.5, CH	108.3, CH
2′	83.4, CH	83.5, CH	82.5, CH
3′	78.7, CH	78.6, CH	78.7, CH
4′	85.5, CH	85.3, CH	84.7, CH
5′	71.0, CH	71.0, CH	71.0, CH
6′	76.1, CH_2_	76.1, CH_2_	74.6, CH_2_
6′OCH_3_	59.4, CH_3_	59.5, CH_3_	58.8, CH_3_
MeGal*_p_*			
1′′	103.4, CH	103.2, CH	102.9, CH
2′′	75.5, CH	75.4, CH	75.5, CH
3′′	88.7, CH	88.6, CH	87.7, CH
4′′	71.8, CH	71.9, CH	71.8, CH
5′′	78.5, CH	78.5, CH	78.5, CH
6′′	63.8, CH_2_	63.8, CH_2_	62.6, CH_2_
3′′-OCH_3_	61.5, CH_3_	61.4, CH_3_	60.4, CH_3_

^1^ Assignments aided by ^1^H–^1^H COSY, TOCSY, HMBC, and NOESY experiments.

**Table 2:** ^1^H NMR spectroscopic data (600 MHz, pyridine-*d*_5_) for hesperusides A-C (**1**–**3**) ^1^ (δ in ppm, *J* in Hz).

Position	1	2	3
1	2.06 m; 1.50 m	2.06 m; 1.50 m	1.52 m; 1.32 m
2	1.85 m; 2.02 m	1.88 m; 2.02 m	1.80 m; 1.21 m
3	4.52 m	4.50 m	4.31 m
4	1.92 m; 2.48 t (14.6, 16.1)	1.92 m; 2.48 t (16.3, 16.3)	1.90 m; 2.31 m
5	2.91 brd (15.0)	2.94 brd (16.2)	2.79 brd (13.0)
6	4.34 m	4.33 m	4.66 m
7	4.96 d (2.2)	4.96 m	3.71 d (2.4)
8			
9	2.80 brt (8.9)	2.80 brt (8.6)	2.64 m
10			
11	1.75 m; 1.74 m	1.76 m; 1.74 m	1.52 m; 1.62 m
12	1.38 m; 1.86 m	1.48 m; 1.96 m	1.32 m; 1.79 m
13			
14			
15	3.10 m; 3.21 m	3.06 m; 3.27 m	2.90 m; 3.10 m
16	4.79 m	4.78 m	4.79 m
17	1.72 d (9.9)	1.72 d (9.9)	1.60 dd (9.6, 2.5)
18	1.06 s	1.06 s	0.91 s
19	1.32 s	1.32 s	1.23 s
20	2.57 m	1.76 m	1.71 m
21	1.24 d (7.9)	1.09 d (7.2)	0.78 d (7.0)
22	6.15 m	1.86 m; 1.60 m	1.38 m
23	5.45 m	1.50 m; 1.26 m	1.90 m; 2.31 m
24	2.09 m	1.27 m	
25	1.68 m	1.58 m	2.42 m
26	0.96 d (5.1)	0.92 d (7.6)	1.11 d (7.6)
27	0.98 d (5.1)	0.92 d (7.6)	1.11 d (7.6)
28			4.75 s; 4.77 s
MeGal*_f_*			
1′	5.80 d (3.2)	5.81 d (1.1)	5.61 d (3.2)
2′	4.76 m	4.73 m	4.56 m
3′	4.87 m	4.86 m	4.87 m
4′	4.70 m	4.69 m	4.54 m
5′	4.48 m	4.46 m	4.48 m
6′	4.03 m	4.00 m	3.80 m
6′-OCH_3_	3.45 s	3.44 s	3.29 s
MeGal*_p_*			
1′′	4.80 d (7.7)	4.81 d (7.6)	4.70 d (7.7)
2′′	3.94 m	3.93 m	3.94 m
3′′	3.76 t	3.74 t	3.58 t
4′′	4.15 m	4.09 m	4.15 m
5′′	3.89 m	3.89 m	3.89 m
6′′	4.37 m; 4.53 m	4.34 m; 4.53 m	4.32 m; 4.73 m
3′′-OCH_3_	3.91 s	3.90 s	3.72 s

^1^ Assignments aided by ^1^H–^1^H COSY, TOCSY, HMQC, HMBC, and NOESY experiments.

**Table 3:** In vitro cytotoxicity (IC_50_: μM) of glycosides **1**–**3** against three tumor cell lines (mean ± S.D., *n* = 3).

Cell Line	1	2	3	HCP ^a^
A-549	3.62 ± 1.08 *	1.84 ± 0.65 *	2.40 ± 0.73 *	0.84 ± 0.05
MOLT-4	2.59 ± 0.94 *	0.68 ± 0.12	2.12 ± 0.81 *	0.16 ± 0.03
BEL-7402	5.26 ± 0.36 *	2.67 ± 0.54 *	5.72 ± 0.82 *	0.03 ± 0.05

^a^ HCP, 10-hydroxycamptothecine, a positive control; * the data was significantly different from the control (*P* < 0.05).

## Supplementary Materials

The MS and NMR data of Compounds **1**–**3** are available online at www.mdpi.com/1660-3397/14/10/189/s1. Figure S1: IR spectrum of hesperuside A (**1**), Figure S2: ^13^C NMR spectrum of hesperuside A (**1**) in pyridine-*d_5_* (150 MHz), Figure S3: ^1^H NMR spectrum of hesperuside A (**1**) in pyridine-*d_5_* (600 MHz), Figure S4: HMQC spectrum of hesperuside A (**1**), Figure S5: ^1^H–^1^H COSY spectrum of hesperuside A (**1**), Figure S6: HMBC spectrum of hesperuside A (**1**), Figure S7: NOESY spectrum of hesperuside A (**1**), Figure S8: TOCSY spectrum of hesperuside A (**1**), Figure S9: EI-MS spectrum of hesperuside A (**1**), Figure S10: HR-EI-MS spectrum of hesperuside A (**1**), Figure S11: IR spectrum of hesperuside B (**2**), Figure S12:. ^1^H NMR spectrum of hesperuside B (**2**) in pyridine-*d_5_* (600 MHz), Figure S13: ^13^C NMR spectrum of hesperuside B (**2**) in pyridine-*d_5_* (150 MHz), Figure S14: HMQC spectrum of hesperuside B (**2**), Figure S15: ^1^H–^1^H COSY spectrum of hesperuside B (**2**), Figure S16: HMBC spectrum of hesperuside B (**2**), Figure S17: NOESY spectrum of hesperuside B (**2**), Figure S18: TOCSY spectrum of hesperuside B (**2**), Figure S19: EI-MS spectrum of hesperuside B (**2**), Figure S20: HR-EI-MS spectrum of hesperuside B (**2**), Figure S21: ^1^H NMR spectrum of hesperuside C (**3**) in pyridine-*d_5_* (600 MHz), Figure S22: ^13^C NMR spectrum of hesperuside C (**3**) in pyridine-*d_5_* (150 MHz), Figure S23: DEPT spectrum of hesperuside C (**3**), Figure S24: HMQC spectrum of hesperuside C (**3**), Figure S25: ^1^H–^1^H COSY spectrum of hesperuside C (**3**), Figure S26: HMBC spectrum of hesperuside C (**3**), Figure S27: EI-MS spectrum of hesperuside C (**3**).

## Abbreviations

The following abbreviations are used in this manuscript:

HPLC	High performance liquid chromatography
NMR	Nuclear magnetic resonance spectroscopy
DEPT	Distortionless enhancement by polarization transfer
COSY	Two dimensional ^1^H correlation
HMQC	Heteronuclear multiple-quantum correlation
HMBC	^1^H-detected heteronuclear multiple-bond correlation
NOESY	Nuclear overhauser effect spectroscopy
GC–MS	Gas chromatography-mass spectrometer
TLC	Thin layer chromatography
SRB	Sulforhodamine B
