# Supplementary material for: Three New Cytotoxic Polyhydroxysteroidal Glycosides from Starfish Craspidaster hesperus
Source: Mar Drugs. 2016 Oct 19;14(10):189. doi: 10.3390/md14100189 (PMC5082337; doi:10.3390/md14100189)
Supplement: Supplementary file 1 [file marinedrugs-14-00189-s001.pdf]

Supplementary Materials: Three New Cytotoxic Polyhydroxysteroidal Glycosides from the Starfish *Craspidaster hesperus*

Jun-Xia Kang, Ying-Feng Kang and Hua Han

List of Supporting Information **Page**

Figure S1. IR spectrum of hesperuside A (**1**) S2
Figure S2. ^{13}C NMR spectrum of hesperuside A (**1**) in pyridine-*d*$_5$, 150MHz S2
Figure S3. ^{1}H NMR spectrum of hesperuside A (**1**) in pyridine-*d*$_5$, 600MHz S3
Figure S4. HMQC spectrum of hesperuside A (**1**) S3
Figure S5. ^{1}H-^{1}H COSY spectrum of hesperuside A (**1**) S4
Figure S6. HMBC spectrum of hesperuside A (**1**) S4
Figure S7. NOESY spectrum of hesperuside A (**1**) S5
Figure S8.TOCSY spectrum of hesperuside A (**1**) S5
Figure S9. EI-MS spectrum of hesperuside A (**1**) S6
Figure S10. HR-EI-MS spectrum of hesperuside A (**1**) S6
Figure S11. IR spectrum of hesperuside B (**2**) S7
Figure S12. ^{1}H NMR spectrum of hesperuside B (**2**) in pyridine-*d*$_5$, 600MHz S7
Figure S13. ^{13}C NMR spectrum of hesperuside B (**2**) in pyridine-*d*$_5$, 150MHz S8
Figure S14. HMQC spectrum of hesperuside B (**2**) S8
Figure S15. ^{1}H-^{1}H COSY spectrum of hesperuside B (**2**) S9
Figure S16. HMBC spectrum of hesperuside B (**2**) S9
Figure S17. NOESY spectrum of hesperuside B (**2**) S10
Figure S18.TOCSY spectrum of hesperuside B (**2**) S10
Figure S19. EI-MS spectrum of hesperuside B (**2**) S11
Figure S20. HR-EI-MS spectrum of hesperuside B (**2**) S11
Figure S21. ^{1}H NMR spectrum of hesperuside C (**3**) in pyridine-*d*$_5$, 600MHz S12
Figure S22. ^{13}C NMR spectrum of hesperuside C (**3**) in pyridine-*d*$_5$, 150MHz S12
Figure S23. DEPT spectrum of hesperuside C (**3**) S13
Figure S24. HMQC spectrum of hesperuside C (**3**) S13
Figure S25. ^{1}H-^{1}H COSY spectrum of hesperuside C (**3**) S14
Figure S26. HMBC spectrum of hesperuside C (**3**) S14
Figure S27. EI-MS spectrum of hesperuside C (**3**) S15

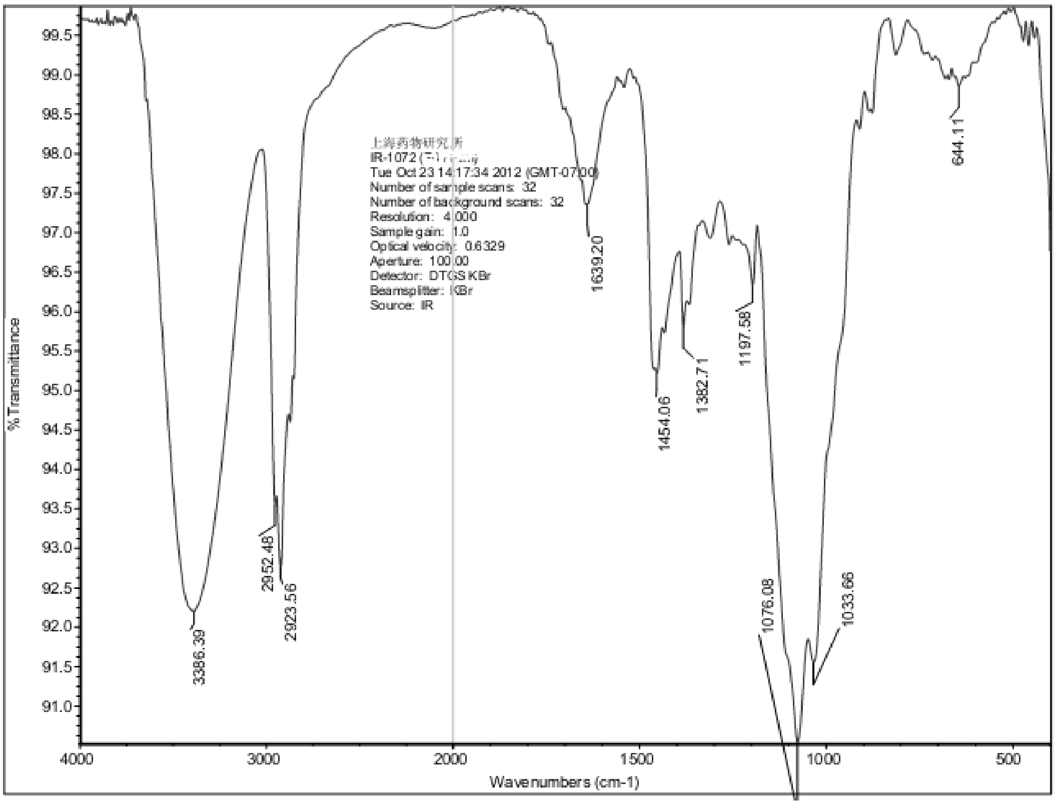

Figure S1. IR spectrum of hesperuside A (**1**).

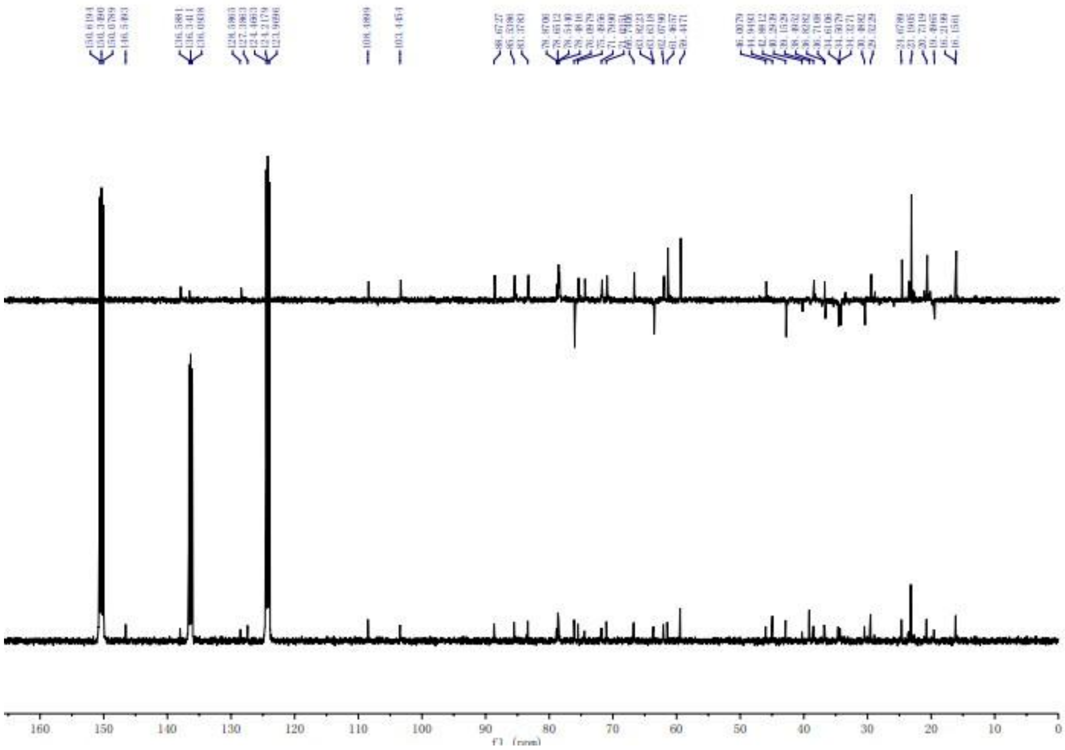

Figure S2. ^{13}C NMR and DEPT spectrum of hesperuside A (**1**) in pyridine-d_5, 150MHz.

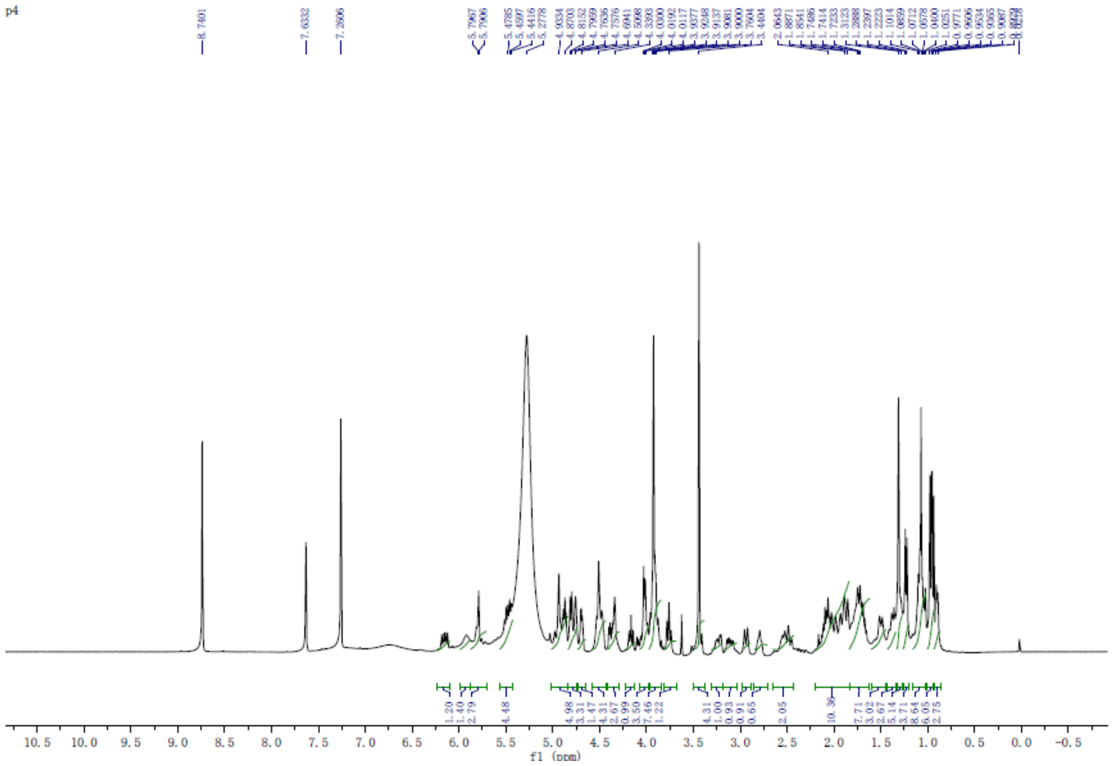

Figure S3. ^{1}H NMR spectrum of hesperuside A (**1**) in pyridine-d_5, 600MHz.

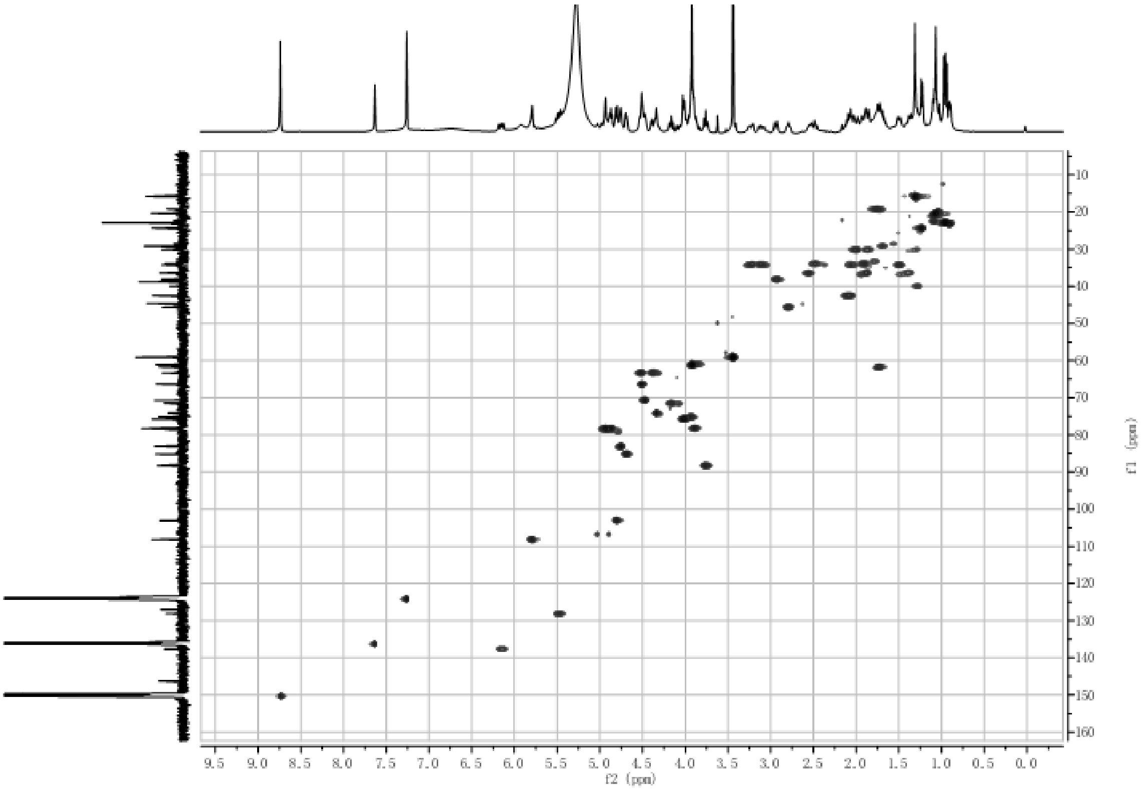

Figure S4. HMQC spectrum of hesperuside A (**1**).

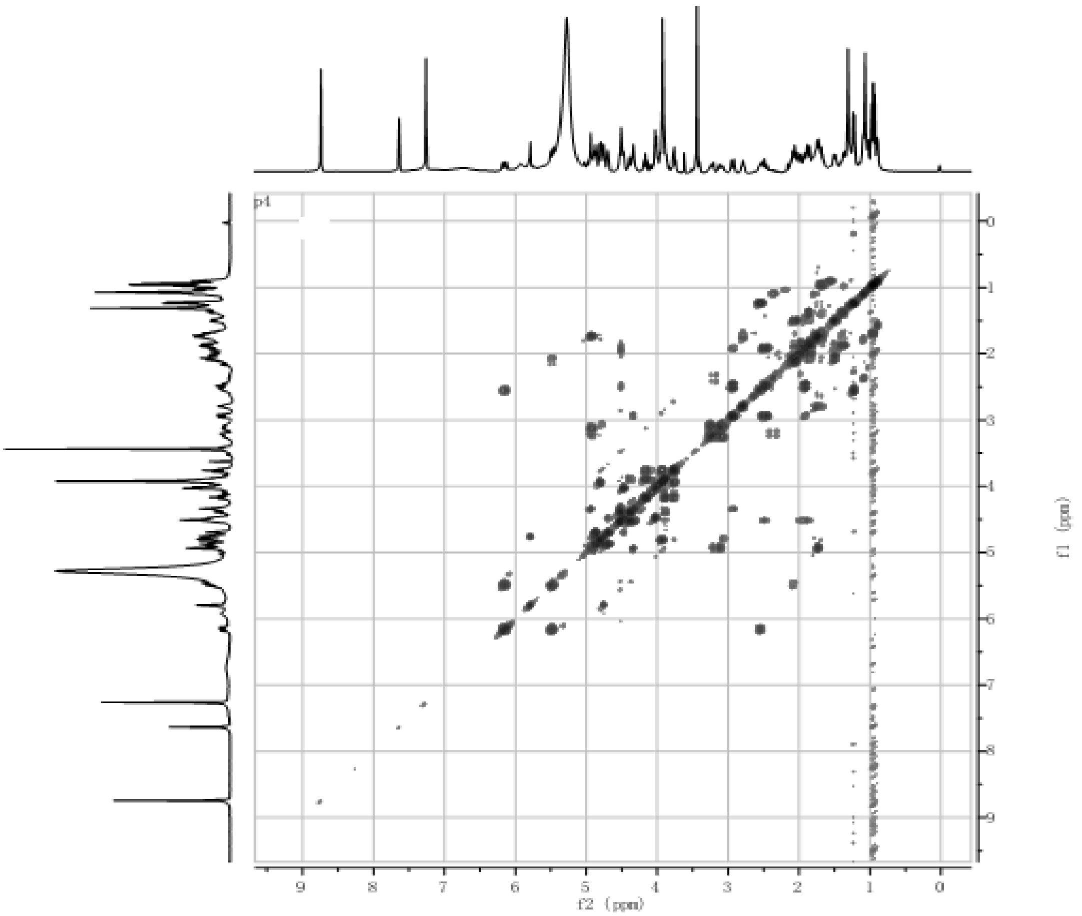

Figure S5. ^{1}H-^{1}H COSY spectrum of hesperuside A (**1**).

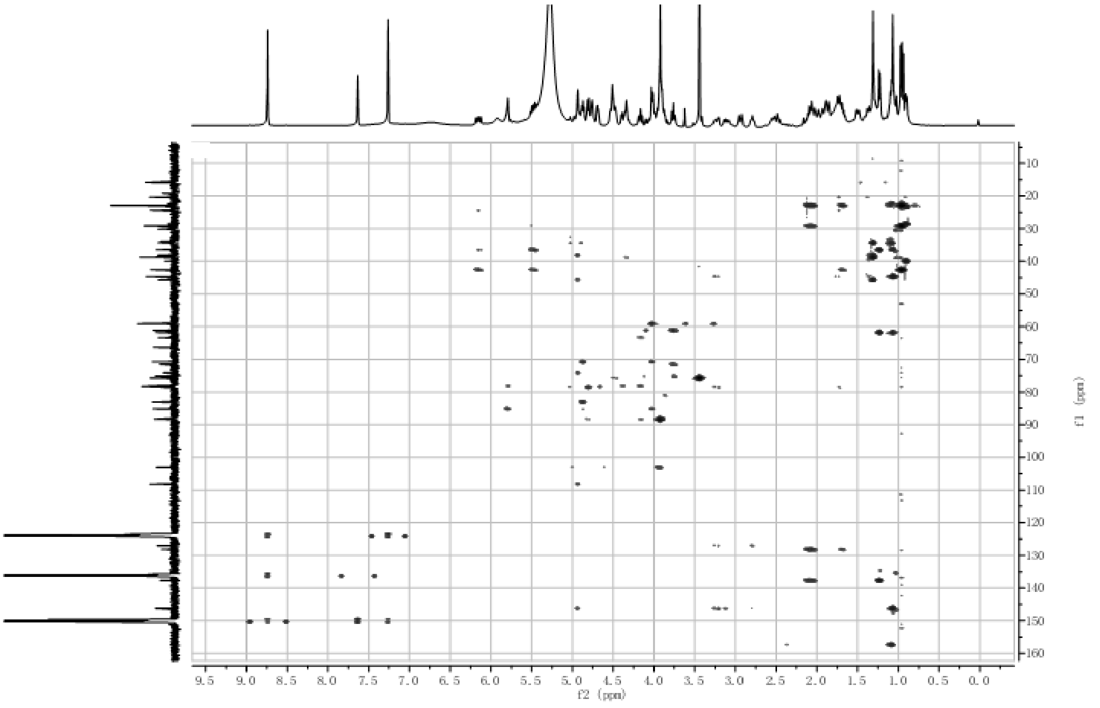

Figure S6. HMBC spectrum of hesperuside A (**1**).

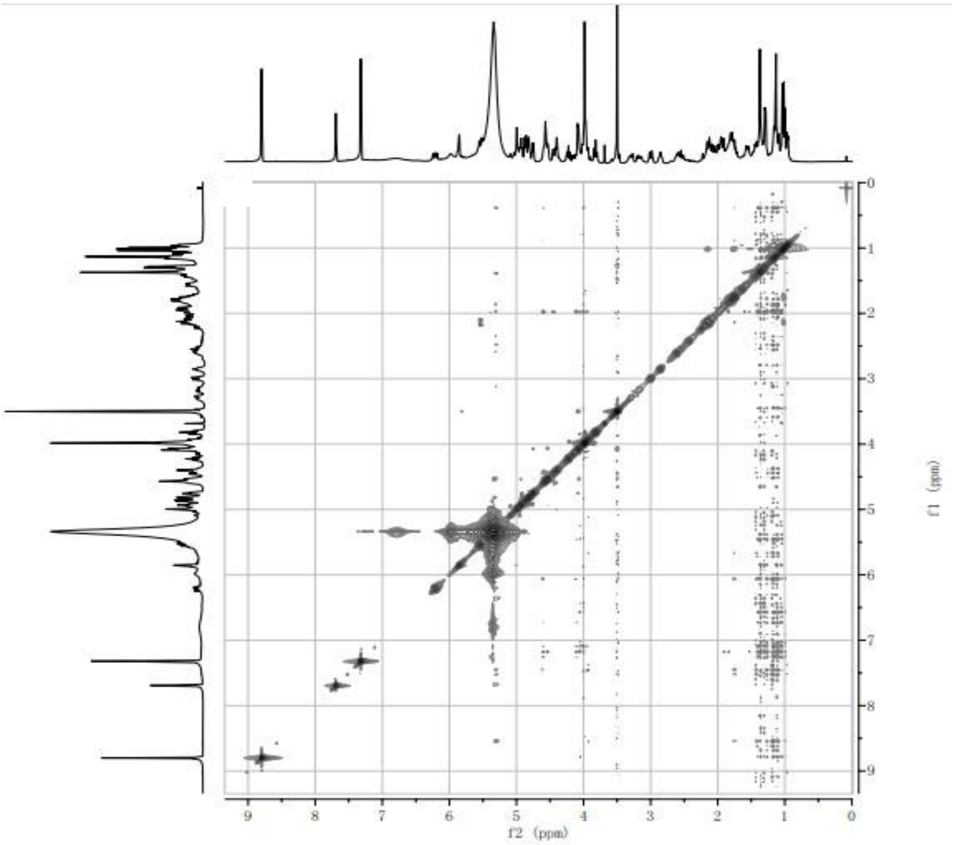

Figure S7. NOESY spectrum of hesperuside A (**1**).

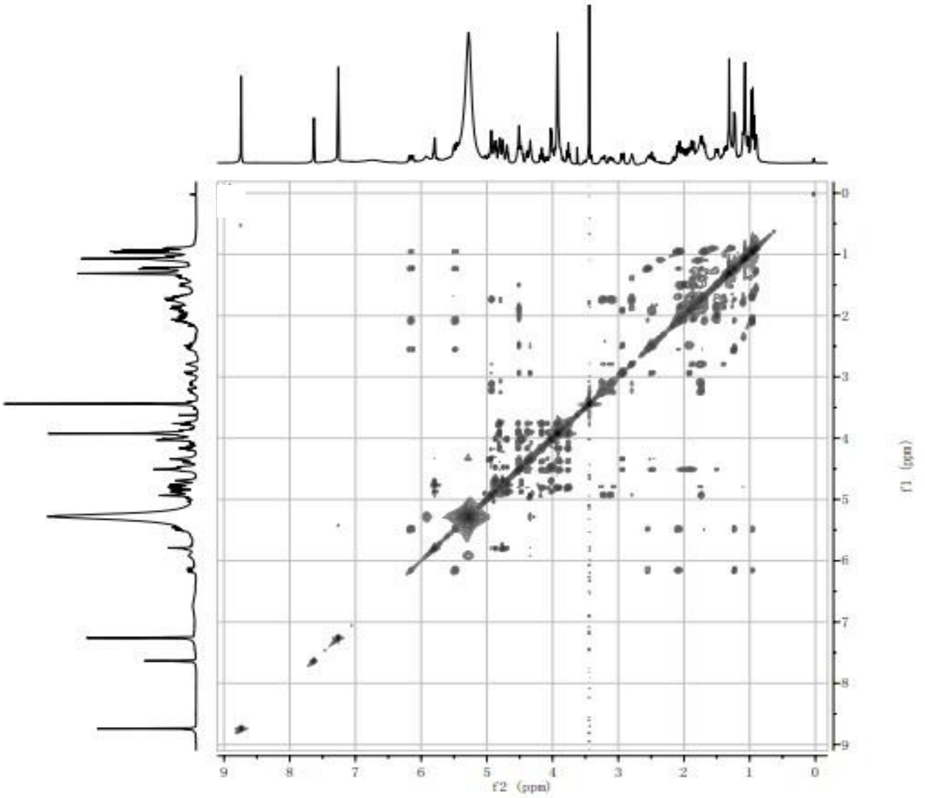

Figure S8.TOCSY spectrum of hesperuside A (**1**).

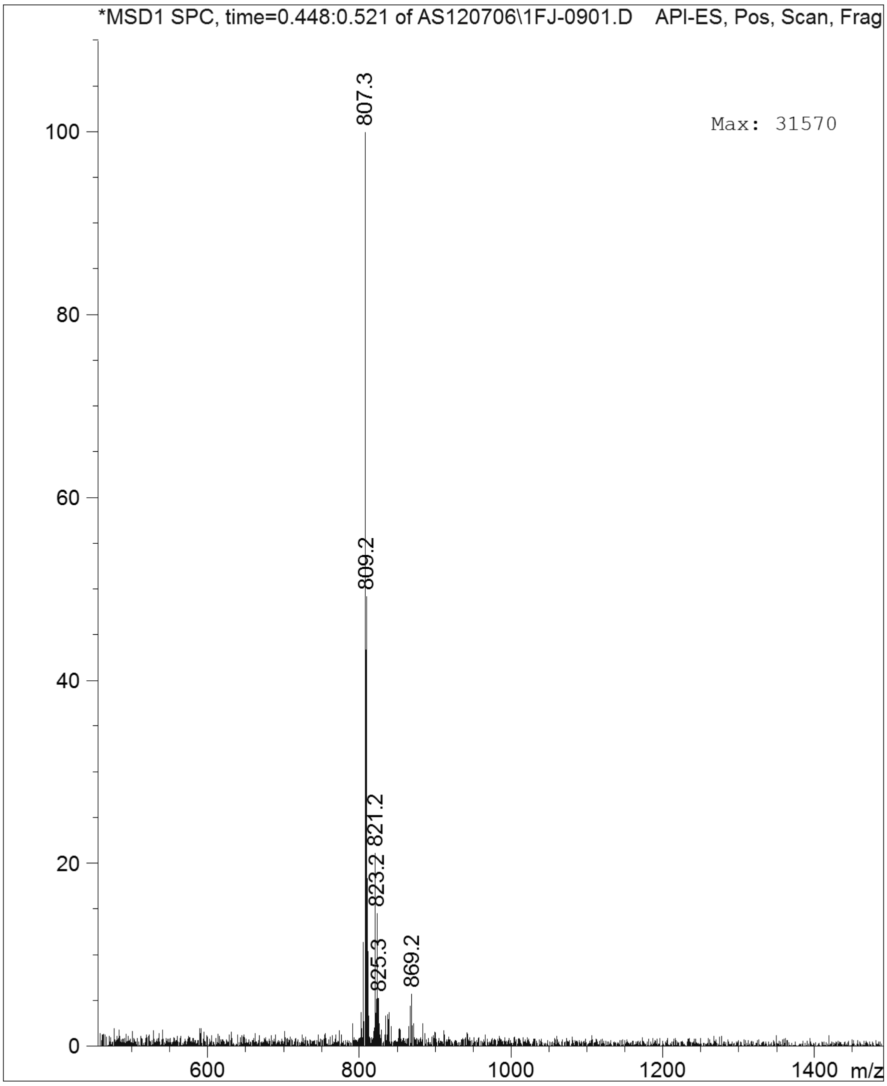

Figure S9. EI-MS spectrum of hesperuside A (**1**).

Ion Mass (Measured) 807.45341

Sum Formula	Sigma	m/z	Err [ppm]	Mean Err [ppm]	Err [mDa]	rdb	N Rule	e^-
C 44 H 66 N 1 Na 1 O 11	0.088	807.45281	-0.75	-1.70	-0.61	12.00	ok	odd
C 41 H 68 Na 1 O 14	0.091	807.45013	-4.07	-5.62	-3.29	7.50	ok	even

Figure S10. HR-EI-MS spectrum of hesperuside A (**1**).

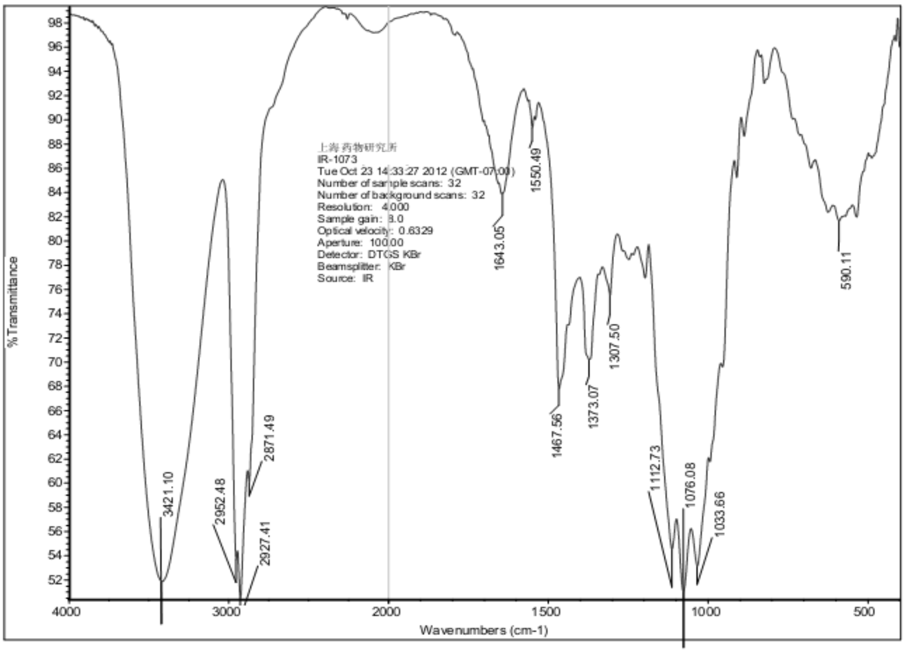

Figure S11. IR spectrum of hesperuside B (**2**).

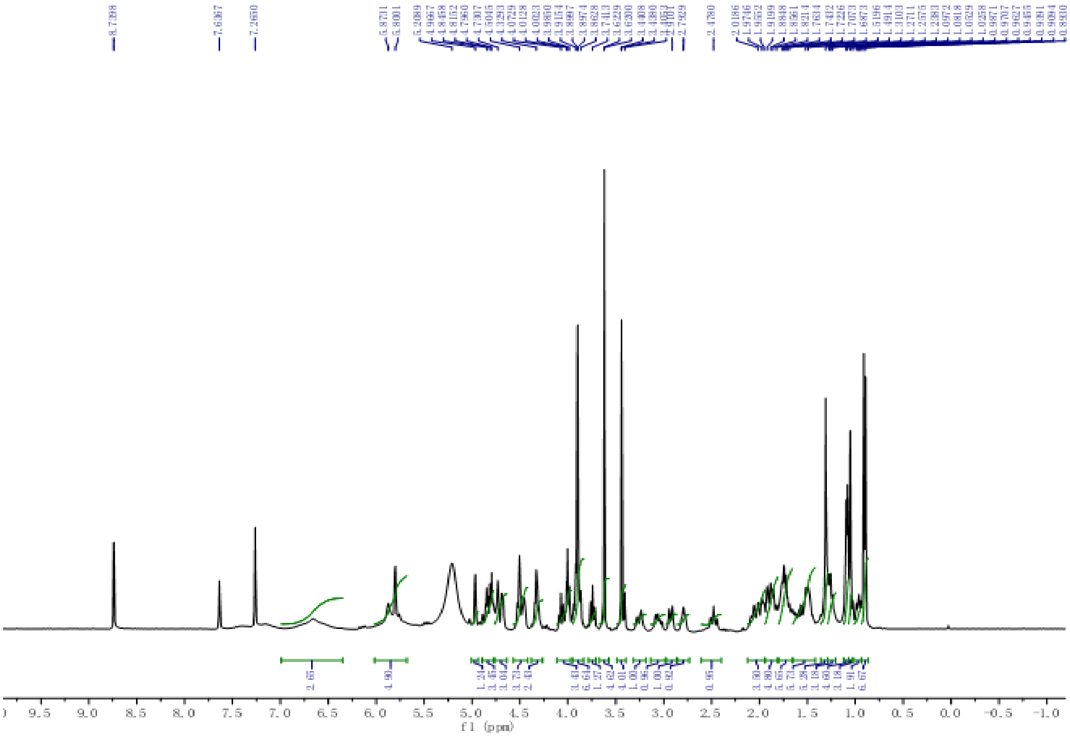

Figure S12. ^{1}H NMR spectrum of hesperuside B (**2**) in pyridine-d_5, 600MHz.

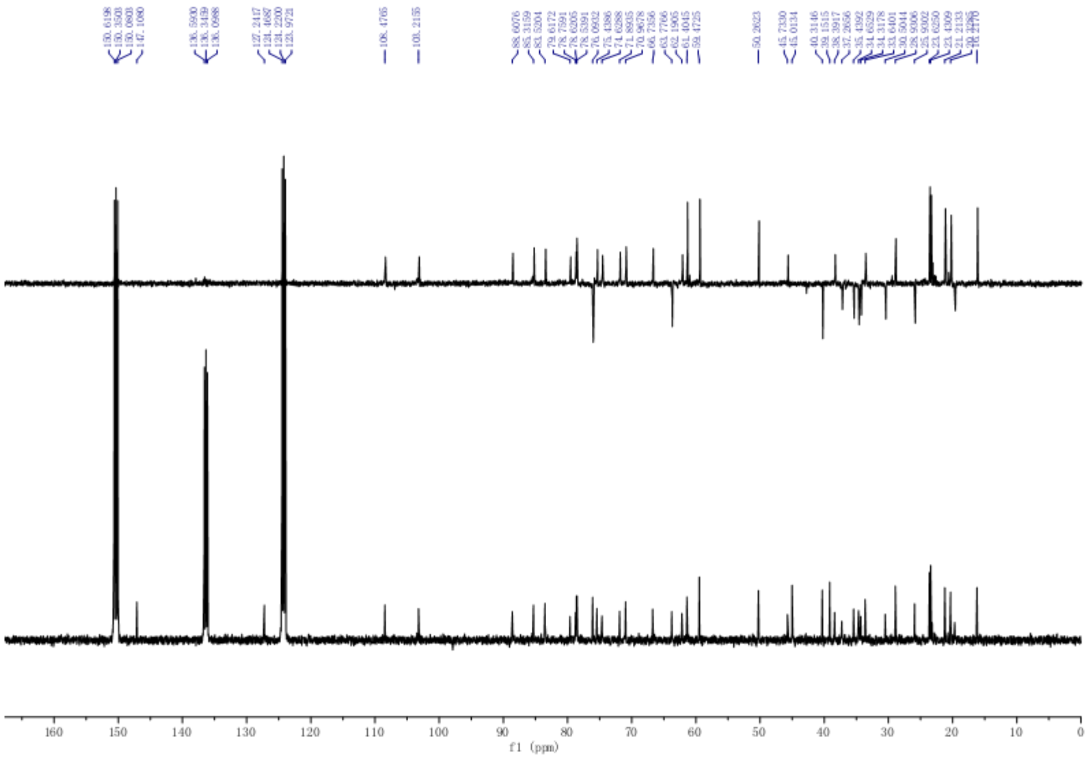

Figure S13. ^{13}C NMR and DEPT spectrum of hesperuside B (**2**) in pyridine-d_5, 150MHz.

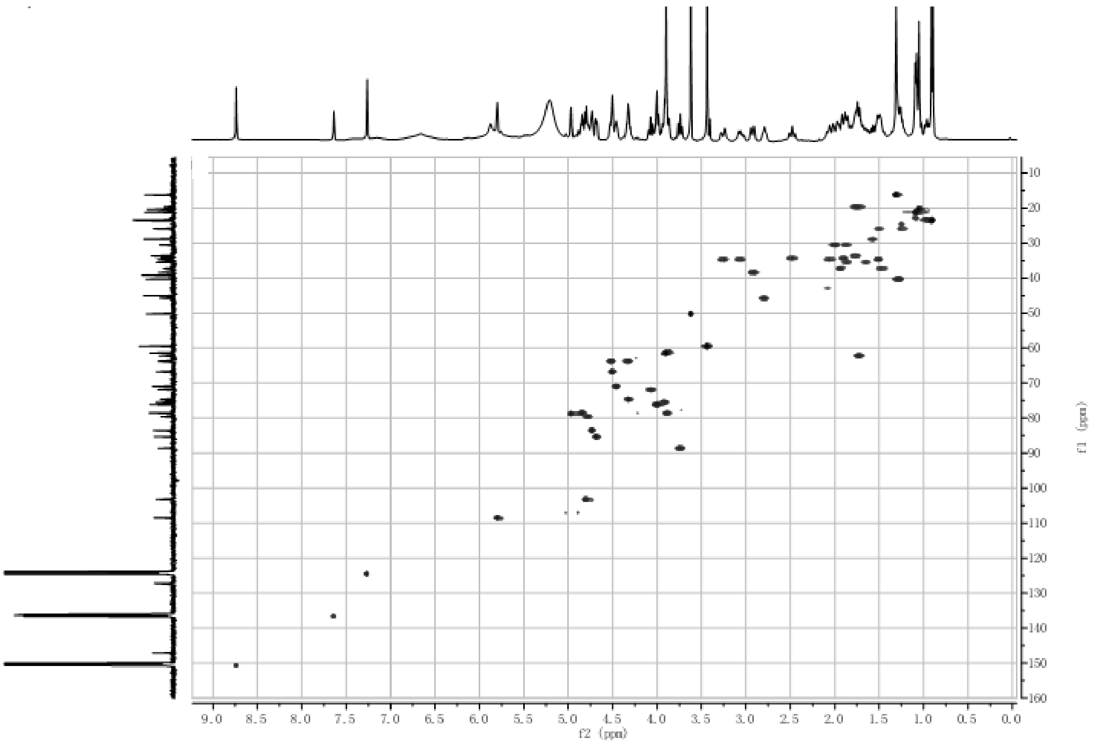

Figure S14. HMQC spectrum of hesperuside B (**2**).

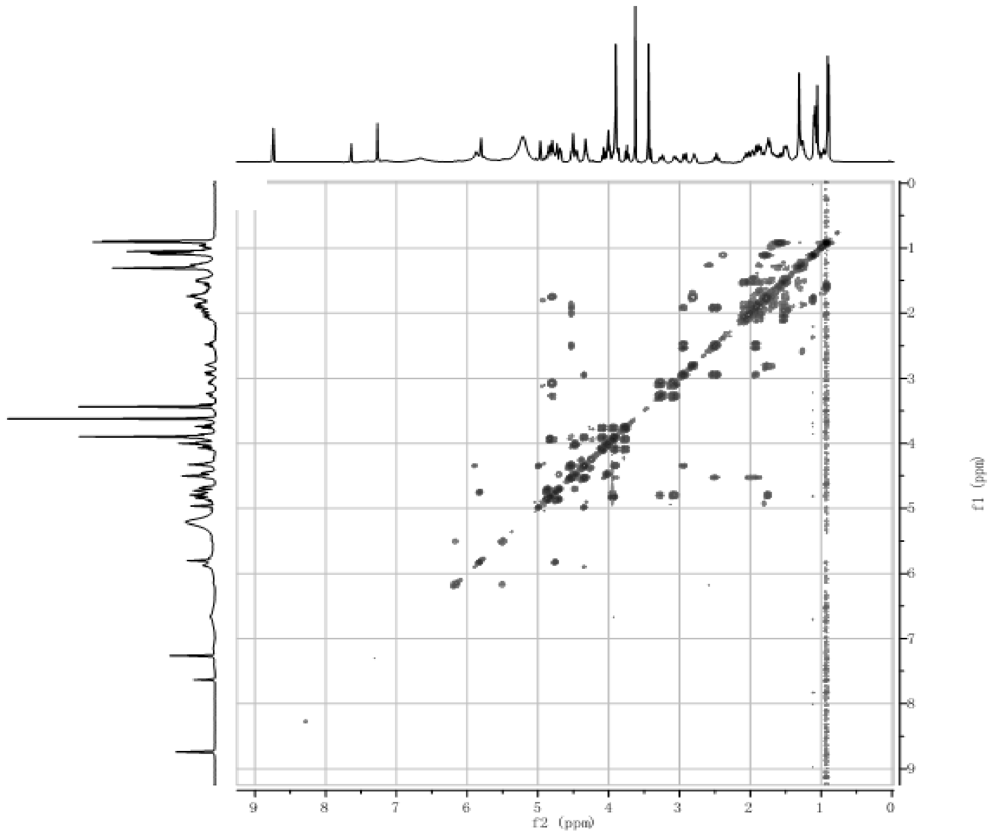

Figure S15. ^1H-^1H COSY spectrum of hesperuside B (**2**).

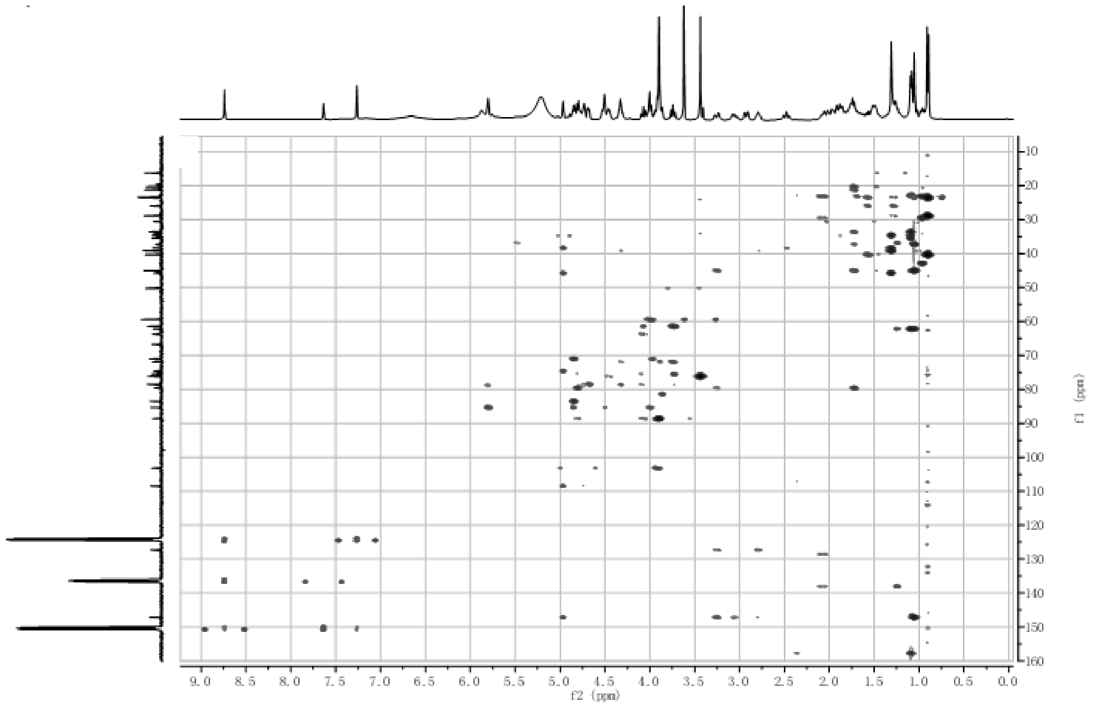

Figure S16. HMBC spectrum of hesperuside B (**2**).

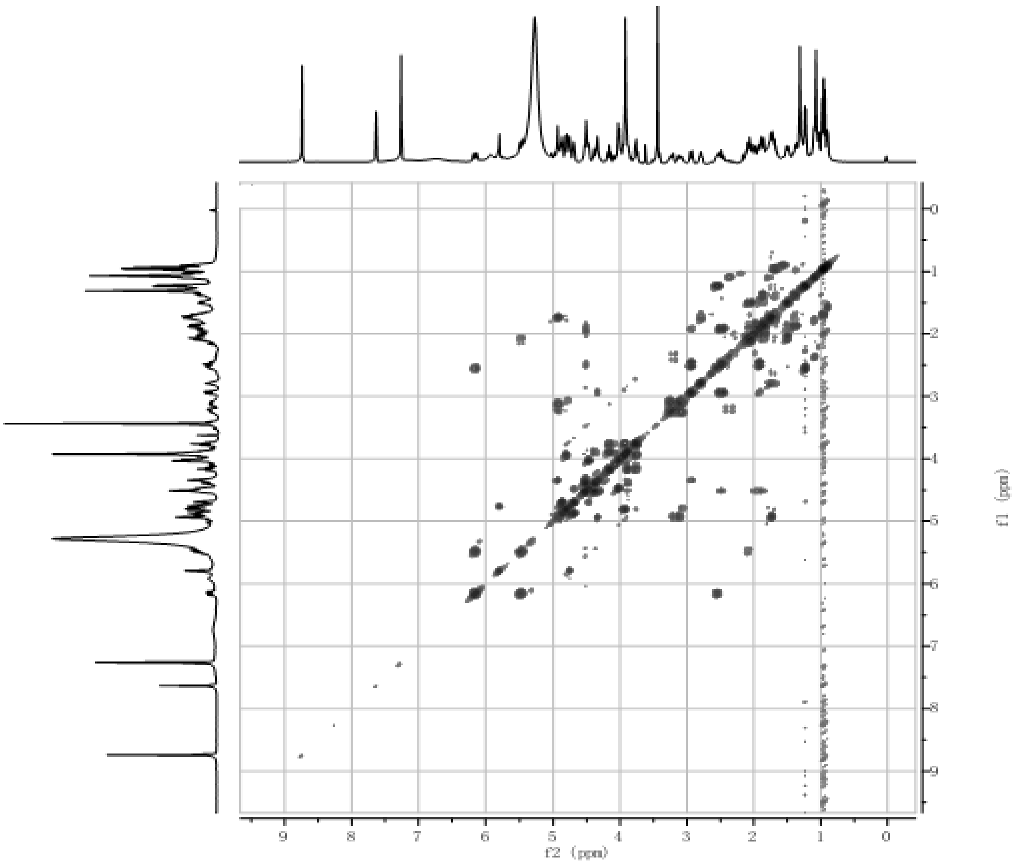

Figure S17. NOESY spectrum of hesperuside B (**2**).

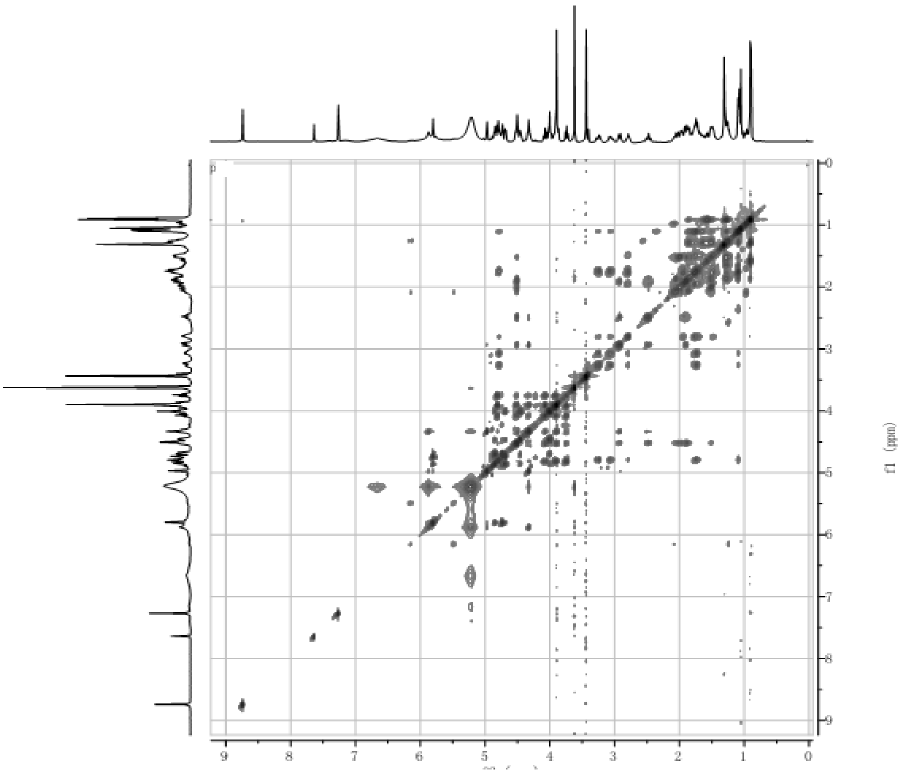

Figure S18.TOCSY spectrum of hesperuside B (**2**).

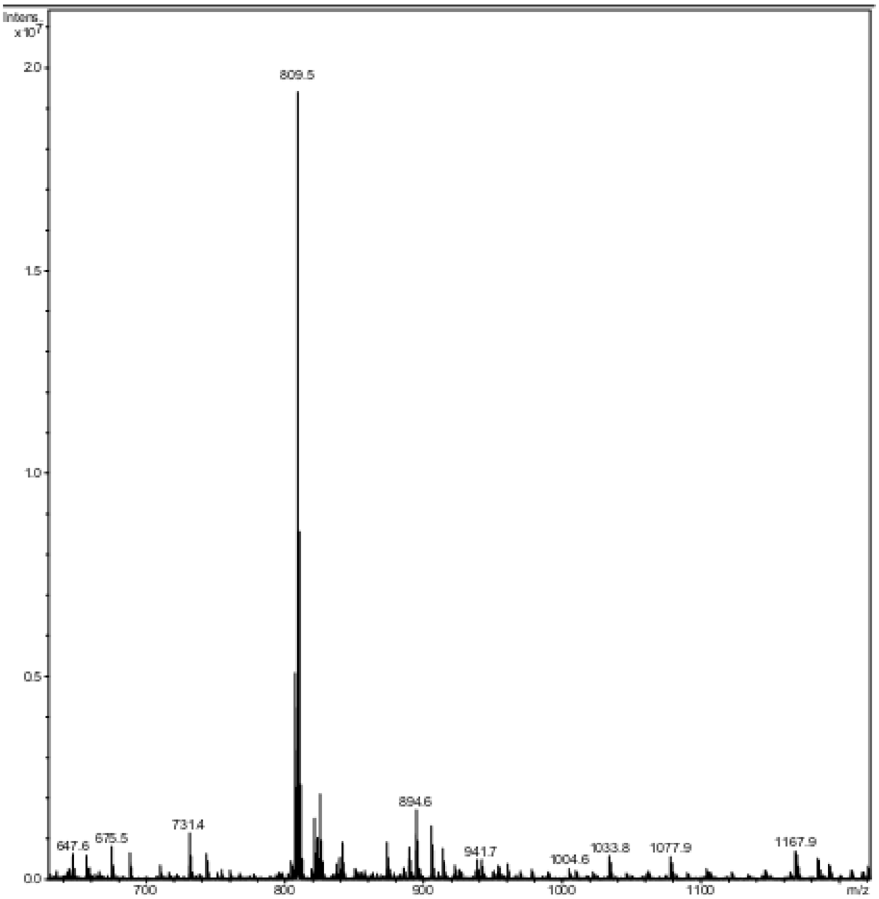

Figure S19. EI-MS spectrum of hesperuside B (**2**).

Ionization Mode	ESI-Positive
Ion Mass (Measured)	809.46384

Sum Formula	Sigma	m/z	Err [ppm]	Mean Err [ppm]	Err [mDa]	rdb	N Rule	e^-
C 41 H 70 Na 1 O 14	0.010	809.46578	2.39	2.23	1.94	6.50	ok	even
C 48 H 66 Na 1 O 9	0.049	809.45990	-4.86	-5.06	-3.94	15.50	ok	even
C 51 H 64 N 1 Na 1 O 6	0.068	809.46258	-1.55	-1.79	-1.26	20.00	ok	odd
C 54 H 62 N 2 Na 1 O 3	0.086	809.46526	1.76	1.48	1.42	24.50	ok	even

Figure S20. HR-EI-MS spectrum of hesperuside B (**2**).

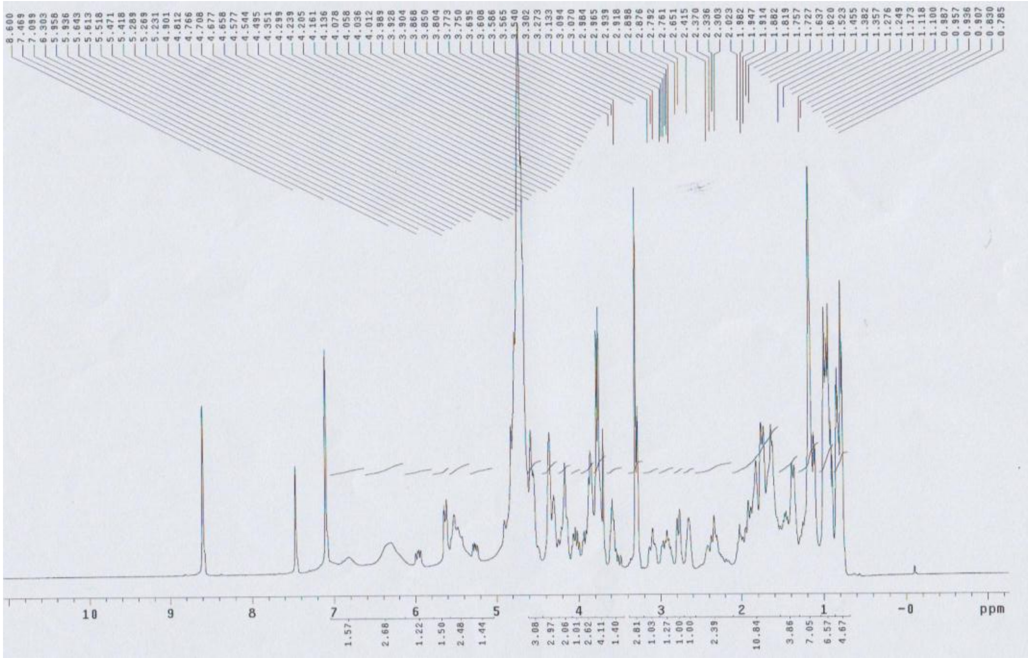

Figure S21. ^{1}H NMR spectrum of hesperuside C (**3**) in pyridine-d_5, 600MHz.

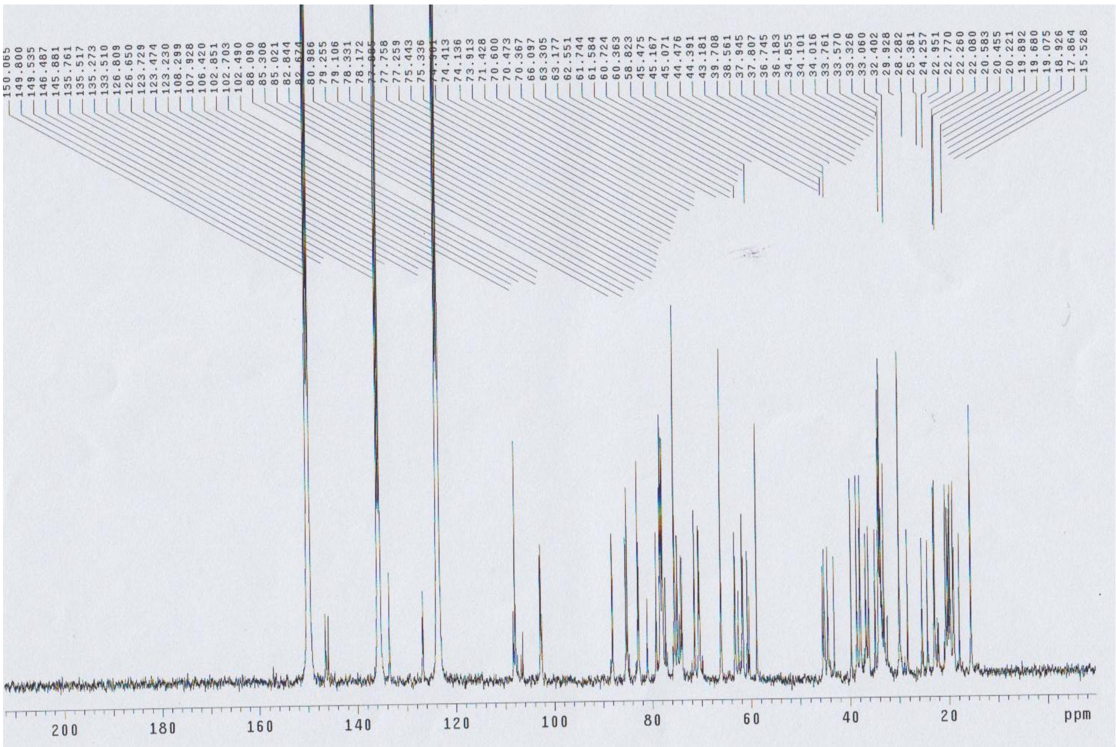

Figure S22. ^{13}C NMR spectrum of hesperuside C (**3**) in pyridine-d_5, 150MHz.

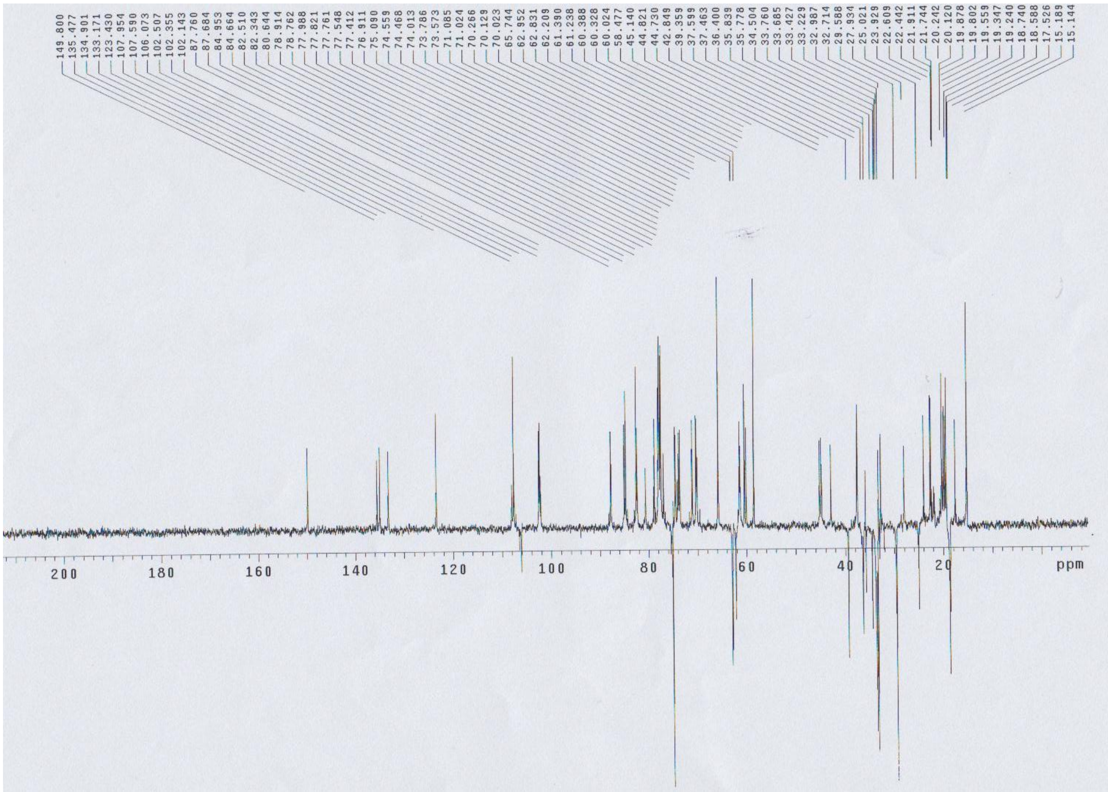

Figure S23. DEPT spectrum of hesperuside C (**3**).

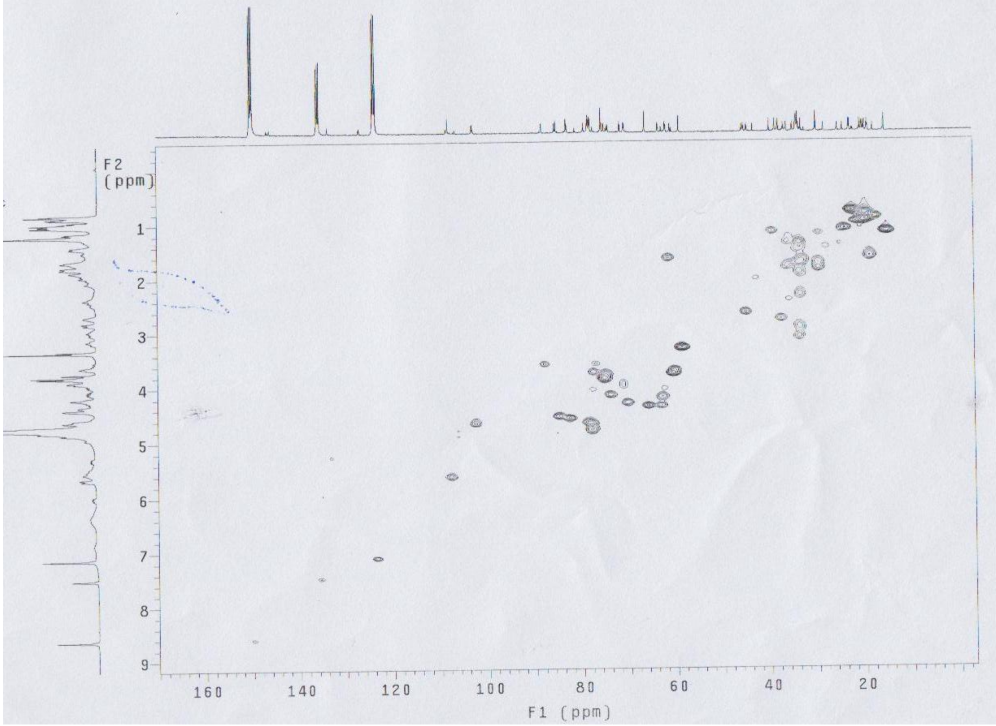

Figure S24. HMQC spectrum of hesperuside C (**3**).

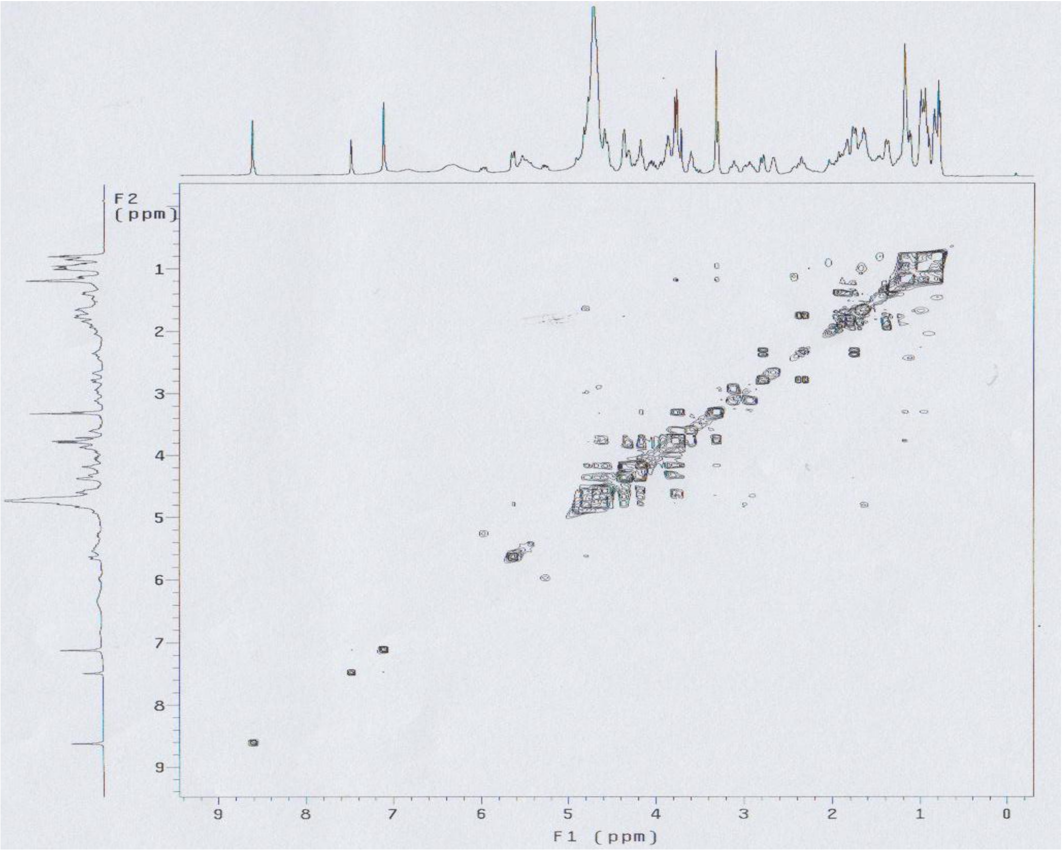

Figure S25. ^{1}H-^{1}H COSY spectrum of hesperuside C (**3**).

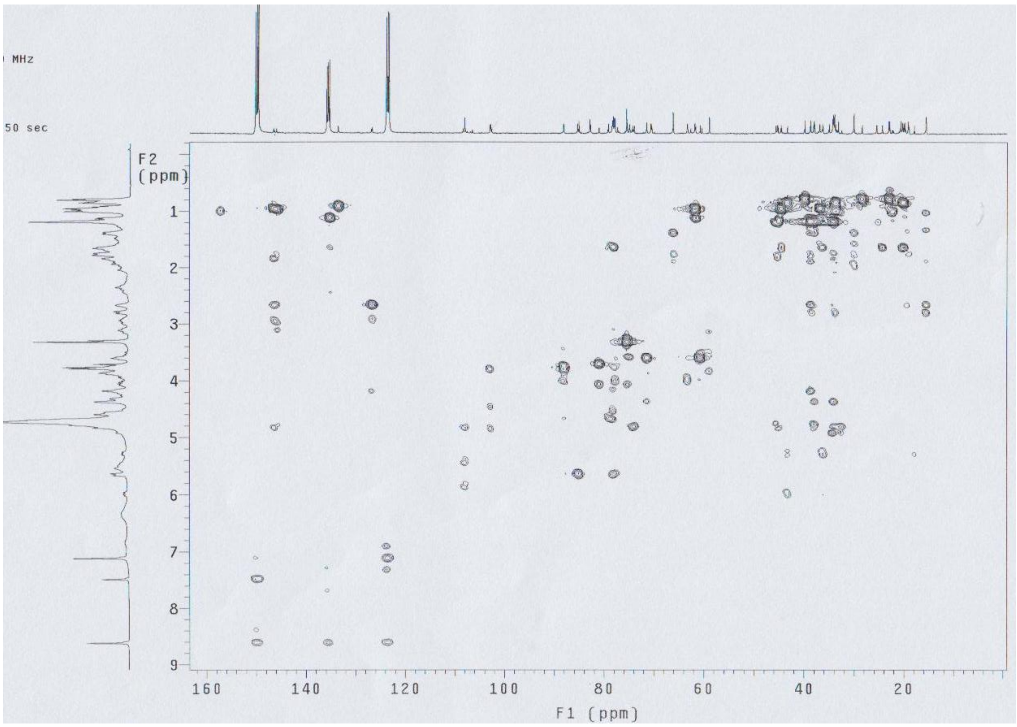

Figure S26. HMBC spectrum of hesperuside C (**3**).

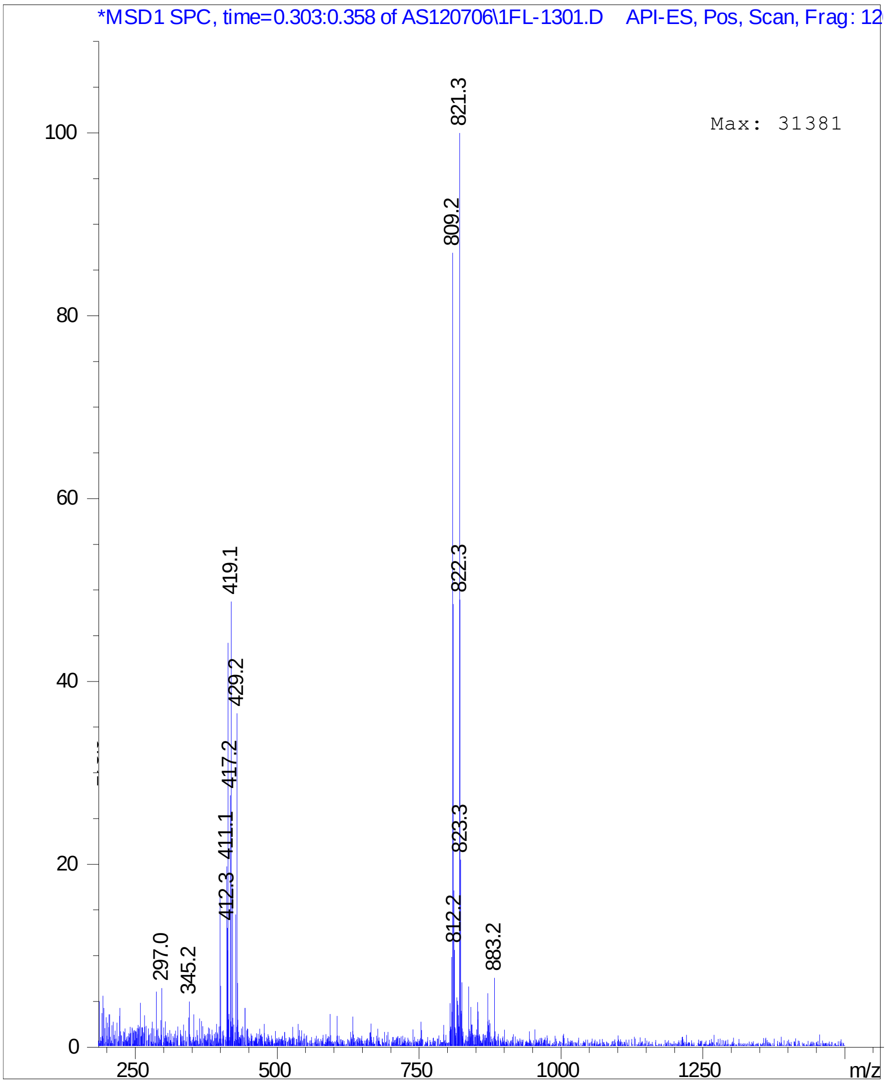

Figure S27. EI-MS spectrum of hesperuside C (**3**).
